# A High Pressure *Operando* Spectroscopy Examination of Bimetal Interactions in ‘Metal Efficient’ Palladium/In_2_O_3_/Al_2_O_3_ Catalysts for CO_2_ Hydrogenation

**DOI:** 10.1002/anie.202312645

**Published:** 2023-09-29

**Authors:** Matthew E. Potter, Sofia Mediavilla Madrigal, Emma Campbell, Lisa J. Allen, Urvashi Vyas, Stephen Parry, Adrián García‐Zaragova, Luis M. Martínez‐Prieto, Pascual Oña‐Burgos, Mads Lützen, Christian D. Damsgaard, Enrique Rodríguez‐Castellón, Nicola Schiaroli, Giuseppe Fornasari, Patricia Benito, Andrew M. Beale

**Affiliations:** ^1^ Chemistry Department University College London 20 Gordon Street London WC1H 0AJ UK; ^2^ UK Catalysis Hub Research Complex at Harwell Rutherford Appleton Laboratory Didcot OX11 0FA UK; ^3^ Cardiff Catalysis Institute School of Chemistry Cardiff University Cardiff CF10 3AT UK; ^4^ Diamond Light Source Rutherford Appleton Laboratory Didcot OX11 0FA UK; ^5^ ITQ, Instituto de Tecnología Química Universitat Politècnica de València-Consejo Superior de Investigaciones Científicas (UPV-CSIC) Av. de los Naranjos S/N 46022 Valencia Spain; ^6^ IIQ, Instituto de Investigaciones Químicas CSIC-Universidad de Sevilla Departamento de Química Inorgánica; Avda Americo Vespucio 49 41092 Seville Spain; ^7^ National Centre for Nanofabrication and Charaterisation Technical University of Denmark Fysikvej Building 307 2800 Kgs. Lyngby Denmark; ^8^ Department of Physics Technical University of Denmark Fysikvej Building 311 2800 Kgs. Lyngby Denmark; ^9^ Departamento de Química Inorgánica Universidad de Málaga Facultad de Ciencias. 29071 Málaga Spain; ^10^ Dipartimento di Chimica Industriale “Toso Montanari” Università di Bologna Alma Mater Studiorum Viale Risorgimento 4 40136 Bologna Italy; ^11^ Center for Chemical Catalysis—C3 Università di Bologna Alma Mater Studiorum Viale Risorgimento 4 40136 Bologna Italy

**Keywords:** CO_2_ Utilization, Catalysis, EXAFS, Operando, Spectroscopy

## Abstract

CO_2_ hydrogenation to methanol has the potential to serve as a sustainable route to a wide variety of hydrocarbons, fuels and plastics in the quest for net zero. Synergistic Pd/In_2_O_3_ (Palldium on Indium Oxide) catalysts show high CO_2_ conversion and methanol selectivity, enhancing methanol yield. The identity of the optimal active site for this reaction is unclear, either as a Pd−In alloy, proximate metals, or distinct sites. In this work, we demonstrate that metal‐efficient Pd/In_2_O_3_ species dispersed on Al_2_O_3_ can match the performance of pure Pd/In_2_O_3_ systems. Further, we follow the evolution of both Pd and In sites, and surface species, under *operando* reaction conditions using X‐ray Absorption Spectroscpy (XAS) and infrared (IR) spectroscopy. In doing so, we can determine both the nature of the active sites and the influence on the catalytic mechanism.

## Graphical abstract

*Operando* spectroscopy under CO_2_ hydrogenation conditions reveals the importance of catalyst design in a metal‐efficient Palladium/In_2_O_3_/Al_2_O_3_ catalyst. Concurrent X‐ray absorption spectroscopy measurements of Pd and In, combined with infrared techniques reveals the roles of the two metals, relating them to the catalytic mechanism with vital insights for future multimetallic catalyst development.

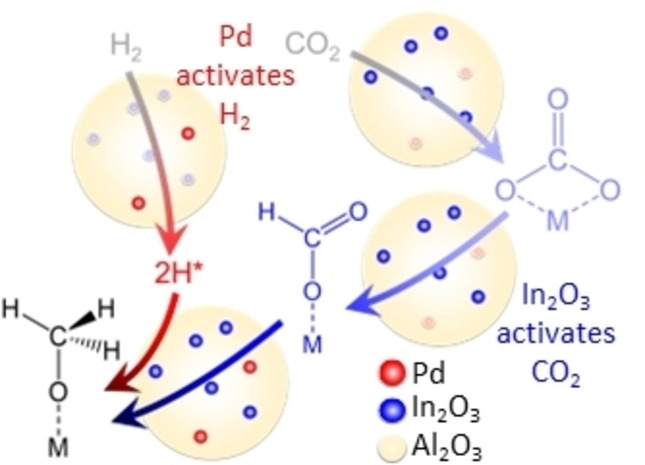

To reach net zero targets, an extra 10 gigatons of CO_2_ must be captured annually,^[1]^ thus, there are many opportunities to utilise CO_2_, such as hydrogenation to methanol.^[2]^ Most literature focuses on the commercial syngas‐to‐methanol Cu/ZnO/Al_2_O_3_ catalyst, the industrial catalyst of choice^[3]^ which suffers from high CO selectivity at high temperatures. Large quantities of Cu are also required (>30 wt%), which are prone to sintering, limiting lifetime.^[4]^ Indium oxide shows promise, with a higher methanol selectivity than commercial Cu/ZnO/Al_2_O_3_.[^4b^, ^5^] Studies suggest this activity comes from oxygen vacancies on In_2_O_3_ surfaces, as identified by a combination of spectroscopic techniques.^[6]^ Despite readily activating CO_2_, In_2_O_3_ has modest activity, struggling to split H_2_ and convert formate (HCOO) to formaldehyde (H_2_CO). Other metals are introduced to improve hydrogen splitting, particularly, combining Pd and In_2_O_3_ improves CO_2_ activity while maintaining high methanol selectivity.^[7]^

Many have investigated the Pd−In_2_O_3_ synergy, debating the occurrence and role of PdIn alloys.[^4a^, ^7^, ^8^] Pd/In_2_O_3_ species, where PdIn was the primary crystalline phase, routinely outperformed monometallic analogues, suggesting a link between PdIn alloys and catalytic activity.^[7b]^ PdIn/SiO_2_ species also show the presence of PdIn and Pd_2_In_3_ phases, depending on the Pd : In ratio, with In Extended X‐ray Absorption Fine Structure (EXAFS) spectroscopy confirming over 80 % of In formed an alloy.^[7c]^ Others demonstrated, with EXAFS, that the synthetic method influences PdIn phase formation, with co‐precipitation creating stable PdIn clusters but dry‐impregnation causing Pd sintering and lower methanol yields.^[4a]^ Similarly, computational work shows that the precise PdIn surface also influences catalytic activity.[^4a^, ^8b^, ^9^] It has been reported PdIn alloys hinder activity, particularly on Pd/In_2_O_3_/SBA‐15, which attiributes decreasing methanol selectivity to PdIn phases. Rui et al. used a peptide‐based method to deposit Pd nanoparticles (NPs) onto In_2_O_3_, where diffraction and XPS (X‐ray Photoelectron Spectroscopy) confirmed alloying, concluding that PdIn alloys hindered Pd's hydrogen splitting ability.[^7a^, ^8d^] Significant debate remains on Pd/In_2_O_3_ systems, where the large In_2_O_3_ quantities encourage alloying, potentially leading to ineffective ‘spectator species’. In our system, we limit the metal quantity, supporting Pd and In_2_O_3_ onto alumina, making a ternary Pd/In_2_O_3_/Al_2_O_3_ species (1 wt% Pd, 12 wt% In_2_O_3_). Al_2_O_3_ was chosen as a robust oxidic support to firmly anchor Pd and In_2_O_3_ to the catalyst. Due to the lower loading in our systems, the formation of alloys will be more evident using EXAFS.^[8d]^ While ex situ and *in situ* studies provide valuable indirect evidence and descriptors for catalytic activity, *operando* spectroscopy identifies active sites in the reaction (Figures S1 to S3 & Table S1).[^6c^, ^10^] This is vital for elevated temperatures with pressures, reactive atmospheres, etc. which can trigger phase changes, and potential alloying.

Our Pd/In_2_O_3_/Al_2_O_3_ contains less In_2_O_3_ than undeposited Pd/In_2_O_3_ but has similar activity (Figure S4), with improved methanol selectivity (Figure S4B, remaining selectivity is CO), making them of significant interest for CO_2_ utilisation.^[11]^ High‐resolution Transmission Electron Microscopy (HRTEM) and Scanning Transmission Electron Microscopy—High‐Angle Annular Dark Field Imaging (STEM‐HAADF) of spent Pd/In_2_O_3_/Al_2_O_3_ show an average particle size of 1.3±0.3 nm and STEM/Electron Energy Loss Spectroscopy (STEM/EELS) maps show regions of overlapping Pd and In, suggesting intimate mixing (Figure S5). XPS of the calcined system suggests (Figure S6) In is present as In_2_O_3_, with and without Pd, with a Pd 3*d*_5/2_ peak at 444.8 eV. Similarly, Pd XPS showed identical signals in Pd/Al_2_O_3_ and Pd/In_2_O_3_/Al_2_O_3_.[^7a^, ^12^] Initial EXAFS data suggests that the Pd exists as PdO, whereas In exists as In_2_O_3_ (Figure 1).

**Figure 1:**
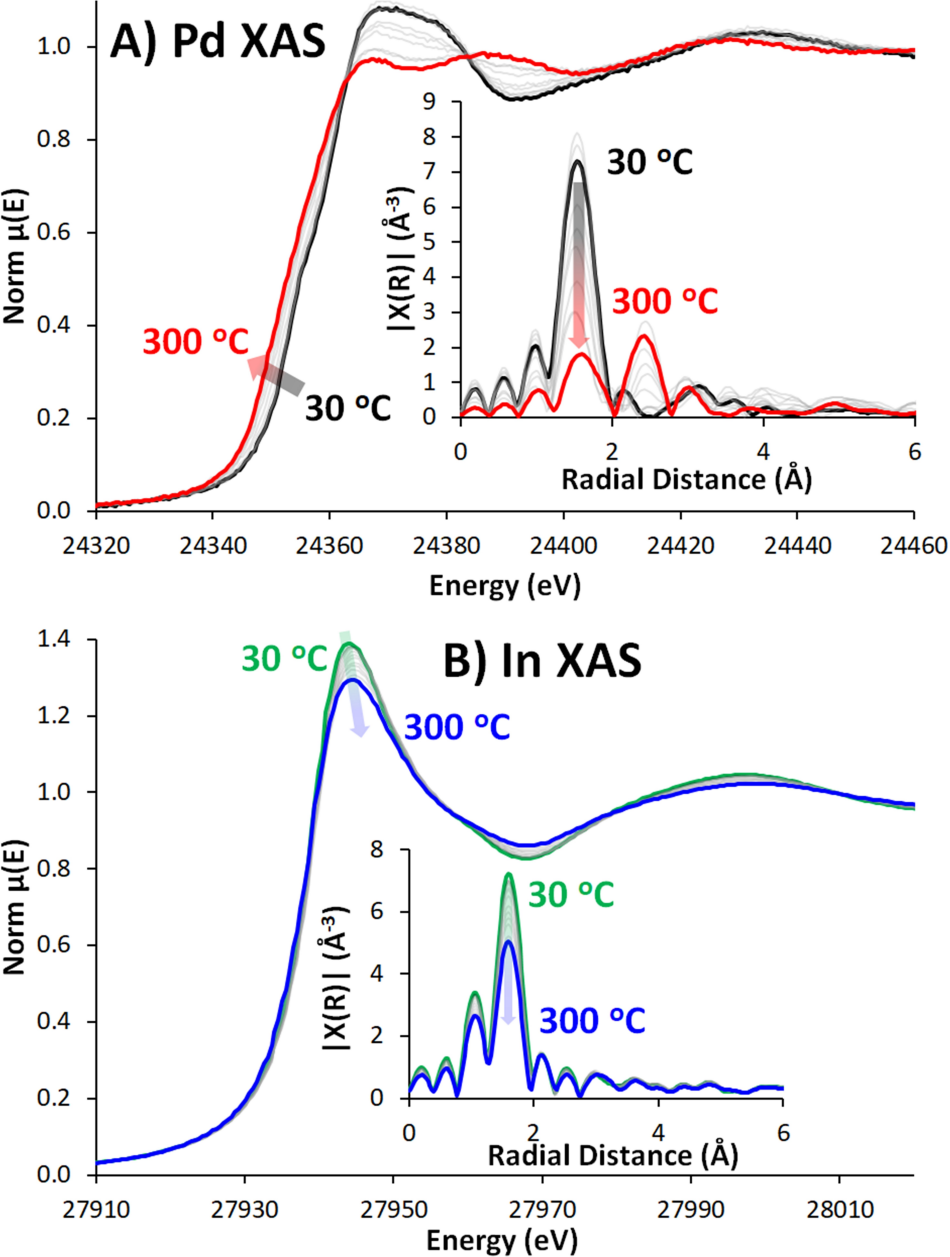
Evolution of Pd and In XAS (X‐ray Absorption Spectroscopy) data of Pd/In_2_O_3_/Al_2_O_3_ during *in situ* reduction, with increasing reduction temperature, showing near‐edge, and insets of R‐space plot for A) Pd K‐edge and B) In K‐edge.

To explore the Pd and In environments, and possible interactions, EXAFS spectra were collected during reduction (Figures 1 & 2), and CO_2_ hydrogenation at 20 bar (Figures 2 & 3). Pd NPs are susceptible to aerial oxidation, so *in situ* and *operando* measurements are needed for genuine insight into active, reactive species. Collecting both In and Pd EXAFS allows changes to be correlated, probing bimetallic interactions. This is complemented by high‐pressure *operando* Diffuse Reflectance Infrared Fourier Transform Spectroscopy (DRIFTS), following similar conditions as the EXAFS. DRIFTS data will help to elucidate the catalytic mechanism, and synergistic enhancement, in the Pd/In_2_O_3_/Al_2_O_3_ system.

**Figure 2:**
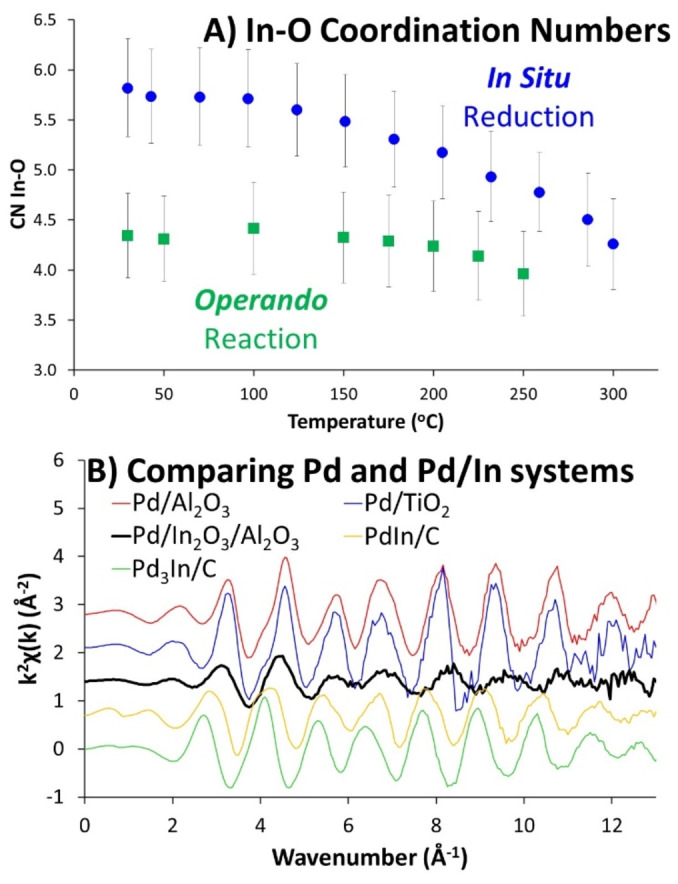
A) Progression of In−O CN during *in situ* reduction and *operando* reaction as temperature increases, and B) Comparing k‐space plots of monometallic and alloyed PdIn systems with our Pd/In_2_O_3_/Al_2_O_3_ species.

EXAFS data (Figure 1A) initially shows PdO, as expected following calcination, transforming to Pd(0) on reduction, with a noticeable decrease in the rising absorption edge intensity and position (Figure S7 & S8).^[13]^ Our XANES (X‐ray Absorption Near‐Edge Structure) data shows the Pd‐edge lowering in energy on reduction, confirming the Pd is being reduced, moving to a similar energy to reduced Pd/Al_2_O_3_ and Pd foil (Figures S7 & S8). R‐space EXAFS shows a decrease in the Pd−O path (1.54 Å radial distance) and an emerging Pd−Pd path at 2.45 Å (Figure 1A). EXAFS and LCF (Linear Combination Fitting) data suggest our system may not be fully reduced, with a small quantity of oxidic character remaining (Figures 1A & S8). Given the small size of our Pd systems (1.3 nm), it is possible the remaining PdO character is due to interactions between surface metallic Pd atoms and the oxidic Al_2_O_3_, or In_2_O_3_ components. If so, such species are unlikely to be accessible to our reagents. The *in situ* Pd reduction EXAFS data was not fitted, as the combination of changing temperature (varying Debye–Waller factors), with varying amounts of two different Pd environments (metallic and oxidic) means many variables must be calculated. Attempts to do this led to a significant increase in error estimation but simply supported the findings already observed by the XANES findings (Figures 1A, S7 & S8) that there was an increase in metallic character and a reduction in oxidic phase.

Initially, In exists as In_2_O_3_. As temperature increases, the oscillations dampen, and the pre‐edge energy slightly decreases (Figures 1B & S9).^[6c]^ LCF analysis suggests a small quantity of In is reduced, though the position of the rising absorption edge shows no discernible movement, suggesting no significant redox occurs (Figure S10). R‐space plots show a strong In−O signal (Figure 1B), which decreases with increased temperature. Notably, there is no evidence of a metallic In−In signal (≈3.0 Å) or In−Pd signal (≈2.5 Å).^[7c]^ The lack of In−In signal (Figure S11, Table S2) is jointly attributed to In_2_O_3_ existing as small nanoparticles, and the amorphous nature of the In_2_O_3_ in our system, as seen by powder X‐ray diffraction (Figure S11B). Both factors limit long‐range order and crystallinity for In_2_O_3_, leading to greatly reduced intensity for subsequent shells. This is likely different to pure In_2_O_3_ or binary Pd/In_2_O_3_ systems, where there is significantly more In_2_O_3_, leading to larger particle sizes, that typically adopt a crystalline bixbyite structure.^[4a]^ The absence of an In−Pd signal in the In EXAFS further suggests the 2.45 Å signal in the Pd EXAFS is Pd−Pd, and not Pd−In, as Pd−In signals should be expected in both Pd and In EXAFS data.

The decreasing In−O signal is likely from oxygen vacancies forming, aligning with the CN (Coordination Number) decreasing from 5.8 (30 °C) to 4.3 (300 °C; Figure 2A & Table S2), and also possibly from increasing thermal disorder. The strong correlation between the Debye–Waller factor (thus, temperature) and coordination number is a constant challenge in EXAFS fitting. To eliminate thermal effects influencing the In−O CN, In EXAFS spectra at 30 °C were compared pre‐ and post‐reduction, confirming a reduction of CN beyond error (Figure 2A, Table S3), supporting oxygen vacancy formation. This is further evidenced by the change in pre‐edge energy, implying a decrease in the overall oxidation state (Figure S9). Furthermore, the In−O bond length shortens from 2.13 to 2.10 Å (Table S3 & Figure S11A), as In forms stronger bonds with remaining oxygen ions. Thus, PdO reduces to Pd metal, whilst In_2_O_3_ forms oxygen vacancies with no significant evidence of alloying.

To investigate possible alloying, EXAFS data of Pd/In_2_O_3_/Al_2_O_3_ was compared with monometallic 1 wt% Pd/Al_2_O_3_ and 5 wt% Pd/TiO_2_ species, along with alloyed bimetallic PdIn/C and Pd_3_In/C species, where the alloying was confirmed with powder XRD (X‐ray Diffraction; Figure S12). The XANES region (Figure S13) shows the EXAFS peaks of Pd/In_2_O_3_/Al_2_O_3_ differ from both monometallic and bimetallic species, though they more closely match the monometallic species. This is reinforced by the k‐space plots (Figure 2B & S14), where the oscillations of the Pd/In_2_O_3_/Al_2_O_3_ species align well with the monometallic species but differ from the bimetallic PdIn/C and Pd_3_In/C species. This provides further evidence that Pd and In, in Pd/In_2_O_3_/Al_2_O_3_, are likely distinct monometallic species. On fitting (Table S3), there was no evidence of a Pd−In feature in the Pd/In_2_O_3_/Al_2_O_3_ system. The alloyed bimetallic species had a notably longer Pd‐Metal (Pd−In here) bond length (both 2.78 Å) than the two monometallic species (2.73 and 2.74 Å). The Pd−M (Pd−Pd) bond length of the Pd/In_2_O_3_/Al_2_O_3_ is smaller than all other systems (2.64 Å) observed, though closer to the monometallic species. While this is shorter than typical Pd−Pd bonds^[14]^ and likely due to their small size. Overall, comparing these model systems suggests that Pd/In_2_O_3_/Al_2_O_3_ does not show significant signs of alloying, providing more evidence that Pd and In_2_O_3_ exist as distinct sites, and not as an alloy.

Post reduction, the cooled system was pressurised to 20 bar. Spectra were collected from 30 to 250 °C (Figure 3), while mass‐spec (MS) data from the outlet confirms methanol concentration (m/z 31, Figure S15) increasing above 200 °C, showing the catalyst performs CO_2_ hydrogenation under *operando* conditions. During the reaction, fitted Pd EXAFS (Table S4) showed there was little change (Figure 3A), confirmed by LCF analysis and the energy position of the rising absorption edge (Figures 2A & S16). R‐space EXAFS showed a subtle variation in the Pd−O and Pd−Pd path intensities (Figure 3A). Changes in CN and bond length were within error (Table S4 & Figures S17 & S18). In also shows little change during the catalytic reaction. The pre‐edge and rising absorption edge energies show minimal variation, whilst there is a subtle dampening of the EXAFS oscillations, typically due to the increase in sample temperature (Figure 3B).

**Figure 3:**
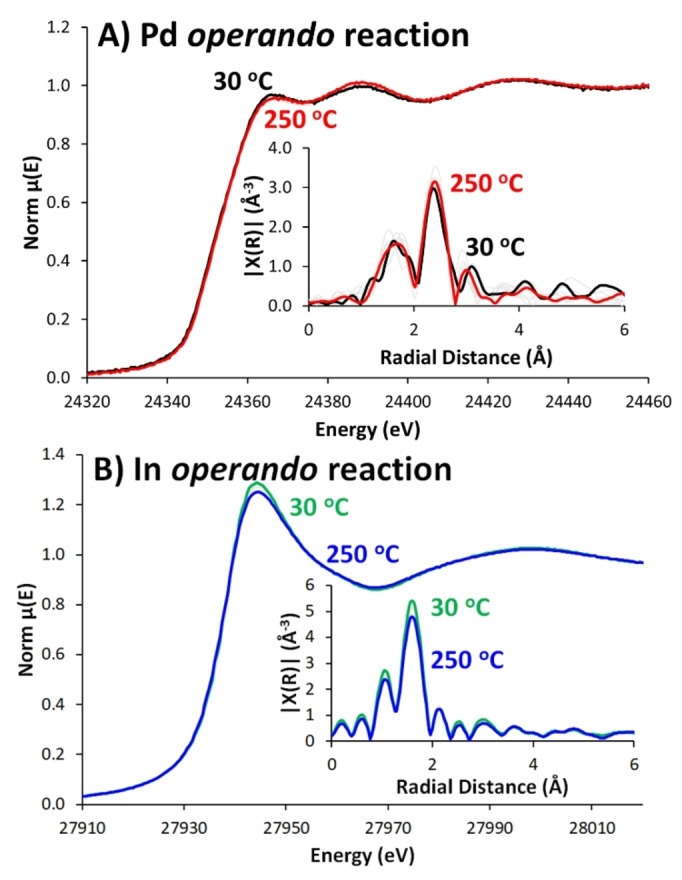
Evolution of Pd and In EXAFS data of Pd/In_2_O_3_/Al_2_O_3_ during *operando* reduction, as a function of increasing reaction temperature, showing XANES progression with insets showing R‐space plot for A) Pd K‐edge and B) In K‐Edge.

LCF analysis shows a slight decrease in In_2_O_3_ character from 50 °C to 250 °C, but there is little correlation with the line energy as temperature changes, suggesting no significant variation in oxidation state (Figure S19). In−O CN changes from 4.3 to 4.0 (Table S5 & Figure 2A), reflecting the decreased intensity in the R‐space plot, but this, and differing In−O bond lengths, are within error (Figure S20). This suggests oxygen vacancies are retained, as the system remains in a primarily reducing atmosphere.

*Operando* EXAFS shows little change from the post‐reduction state, suggesting the state after *in situ* reduction step is a reasonable representation of the actual active species. No significant evidence of alloying, or PdIn phases was found, unlike in Pd/In_2_O_3_ species, where contact between Pd and In_2_O_3_ is ensured. This does, however, not preclude contact between the Pd and In_2_O_3_ phases, or Pd−O−In species in these systems.

*Operando* DRIFTS at 1 and 20 bar explored the Pd−In_2_O_3_ synergy and reaction mechanism (Table S6, Figures S21 to S26). In_2_O_3_/Al_2_O_3_ at 1 bar (Figure S21) shows a rapid loss of polydentate carbonates (1672 cm^−1^) up to 175 °C, coinciding with formate appearance (1606, 1392 and 1305 cm^−1^), and unidentate carbonates (1519 cm^−1^).^[15]^ The C−H region is dominated by formate bands (2994, 2902 and 2740 cm^−1^), with a methoxy shoulder (2923 cm^−1^).^[15]^ At 20 bar (Figure S22), the carbonate region of In_2_O_3_/Al_2_O_3_ is dominated by gaseous rotational bands, though growing formate bands are discernible. At 20 bar, formates still evolve with temperature, but methoxy (2960 & 2852 cm^−1^) and methane formation (3015 cm^−1^ and associated rotational bands) occurs, which is attributed to the DRIFTS cell itself, as these were also seen in an identical experiment with KBr instead of the catalyst.^[15]^ No CO bands were observed, suggesting In_2_O_3_ follows the formate pathway.[^5^, ^6b^] The continually increasing formate signals suggest the rate determining step of the reaction is formate reduction, with In_2_O_3_/Al_2_O_3_ being an inefficient hydrogen activator, due to the continual presence of activated CO_2_ species, and slower formate production, causing lower methanol yields (Figure S4). Pd/Al_2_O_3_ shows CO formation at 1 bar (2030 and broad 1890 cm^−1^, Figure S20) due to Reverse‐Water‐Gas Shift (RWGS). At 1 bar, carbonate species decrease rapidly in Pd/Al_2_O_3_, suggesting improved conversion. The C−H region shows a rapid formate increase until 175 °C, where it decreases as methoxy signals appear (2910 cm^−1^, Figure S23). Aside from rotational bands, the 20 bar and 1 bar Pd/Al_2_O_3_ spectra are similar (Figure S24), except the CO feature decreases with temperature. The C−H region shows the formate signals behaving like the 1 bar system, though signals due to methane (3016 cm^−1^) and methanol are present.^[15]^

Comparing the 1 and 20 bar spectra suggests pressure has little influence on formate formation but improves methoxy and methanol formation. At 1 and 20 bar, the formate species plateau at 175 °C, thus are converted, regardless of pressure. This may be because adsorbed CO_2_ favours RWGS, or more rapid hydrogen activation, readily converting the formate species. Ineffective Pd/Al_2_O_3_ performance is likely due to sluggish CO_2_ activation, as any activated species (formate) are rapidly removed. At 1 bar, Pd/In_2_O_3_/Al_2_O_3_ resembles the In_2_O_3_/Al_2_O_3_ system, with formate continually present, though also some CO (2042 cm^−1^, Figure 4A, S25 & S26). The C−H region shows continuous formate growth, with a discernible methoxy shoulder (2842 cm^−1^) above 250 °C. At 20 bar, Pd/In_2_O_3_/Al_2_O_3_ is again similar to In_2_O_3_/Al_2_O_3_, with a pronounced increase in formate features (1618, 1394 and 1313 cm^−1^), peaking at 250 °C.^[15]^ CO signals are lower than Pd/Al_2_O_3_, suggesting RWGS is less effective, with the formate pathway dominating. This agrees with the C−H stretch region, where formate species transition to methanol and methoxy species, suppressing methane formation. Comparing the mono and bimetallic systems suggests that hydrogen and CO_2_ activations occur more optimally. In_2_O_3_/Al_2_O_3_ continually creates formate, with little methanol or methoxy, as the oxygen vacancies readily activate CO_2_. Pd/Al_2_O_3_ rapidly depleted formate, suggesting hydrogen activation surpassed CO_2_ activation. Pd/In_2_O_3_/Al_2_O_3_ increases formate presence up to 250 °C, and noticeably transitions to strong methanol and methoxy signals (Figures 4B and S28).

**Figure 4:**
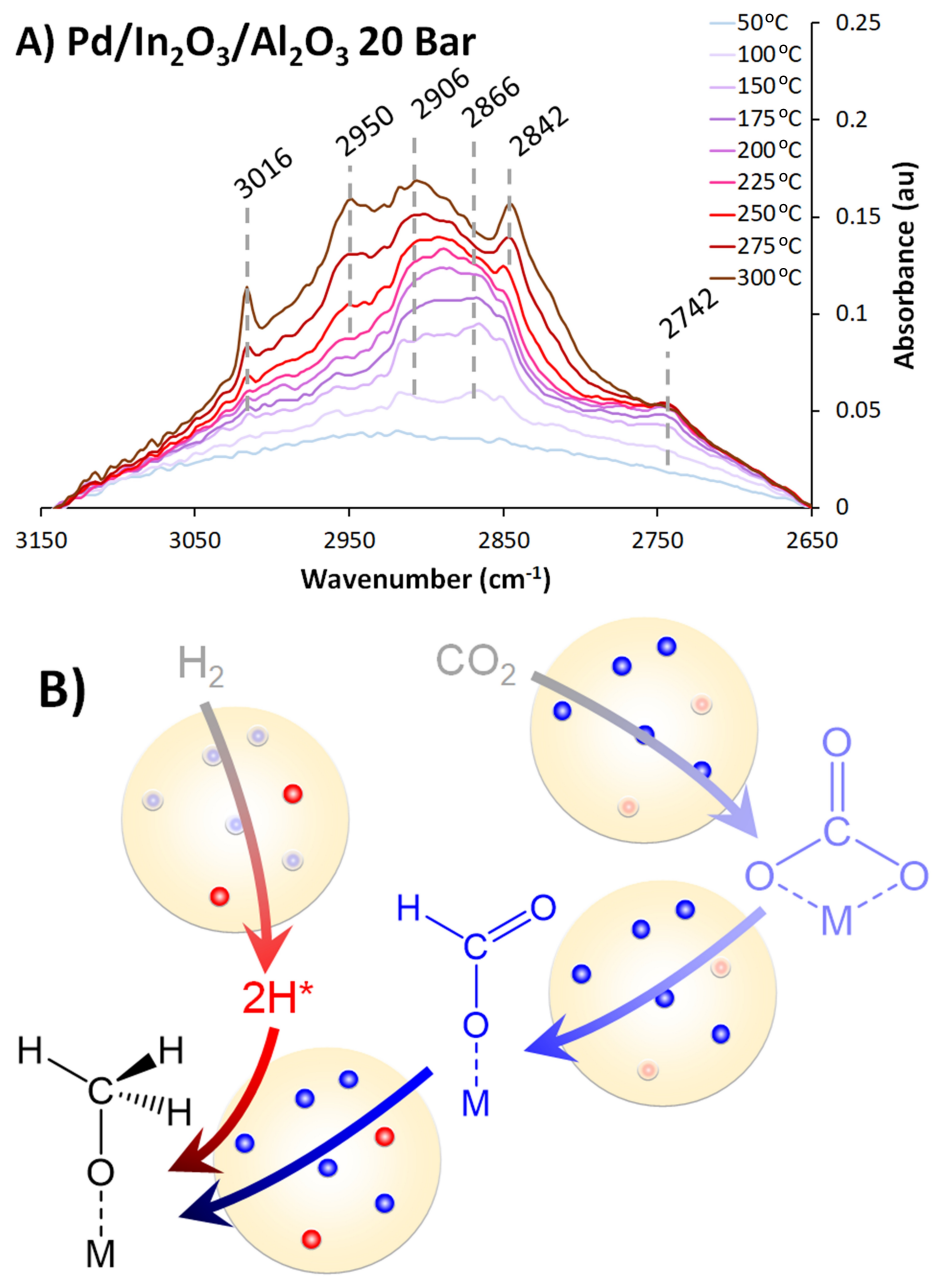
A) *Operando* DRIFTS difference spectra of Pd/In_2_O_3_/Al_2_O_3_, with 30 mL/min of 75 % H_2_ and CO_2_, under 20 Bar of pressure and B) mechanistic implications with Pd represented as red circles, and In_2_O_3_ as blue circles.

Based on the above findings, we suggest that Pd−In synergy in these systems comes from two sites separately performing individual functions (hydrogen and CO_2_ activation, respectively). Subtle changes occur under reaction conditions which may evidence delicate Pd−In_2_O_3_ interactions (i.e., suppressing reverse water gas shift activity), though no *significant* alloying occurs nor appears to be necessary for improved methanol yield (Figure S4). These findings suggest that careful balancing of individual CO_2_ and hydrogen activation processes, and the precise location and interactions of Pd and In_2_O_3_, are key to future catalyst optimisation. Likely extending beyond Pd/In_2_O_3_ systems to other bimetallic CO_2_ hydrogenation systems.

## Conflict of interest

The authors declare no conflict of interest.

## Supporting information

As a service to our authors and readers, this journal provides supporting information supplied by the authors. Such materials are peer reviewed and may be re‐organized for online delivery, but are not copy‐edited or typeset. Technical support issues arising from supporting information (other than missing files) should be addressed to the authors.

Supporting Information

## Data Availability

The data that support the findings of this study are available from the corresponding author upon reasonable request.
