# Supplementary material for: A High Pressure Operando Spectroscopy Examination of Bimetal Interactions in ‘Metal Efficient’ Palladium/In2O3/Al2O3 Catalysts for CO2 Hydrogenation
Source: Angew Chem Int Ed Engl. 2023 Sep 29;62(45):e202312645. doi: 10.1002/anie.202312645 (PMC10952604; doi:10.1002/anie.202312645)
Supplement: Supplementary file 1 — Supporting Information [file ANIE-62-0-s001.pdf]

Angewandte Chemie
Eine Zeitschrift der Gesellschaft Deutscher Chemiker

Supporting Information

A High Pressure *Operando* Spectroscopy Examination of Bimetal Interactions in 'Metal Efficient' Palladium/In_2O_3/Al_2O_3 Catalysts for CO_2 Hydrogenation

M. E. Potter, S. Mediavilla Madrigal, E. Campbell, L. J. Allen, U. Vyas, S. Parry, A. García-Zaragova, L. M. Martínez-Prieto, P. Oña-Burgos, M. Lützen, C. D. Damsgaard, E. Rodríguez-Castellón, N. Schiaroli*, G. Fornasari, P. Benito*, A. M. Beale**

Table of contents

Experimental protocols 2
Catalyst synthesis 2
Catalyst testing of CO_2 hydrogenation to methanol 3
High angle annular dark field/scanning tunnelling electron microscopy (HAADF/STEM) 3
X-ray photoelectron spectroscopy (XPS) 3
Operando X-ray absorption spectroscopy (XAS) 4

Operando diffuse reflectance infrared Fourier transform spectroscopy (DRIFTS) 6
Catalytic data 7
CO_2 hydrogenation to methanol 7
Ex situ characterisation 9
High angle annular dark field scanning tunnelling electron microscopy (STEM-HAADF) 9
X-ray photoelectron spectroscopy (XPS) 10
In situ Reduction XAS data 11
In situ Pd reduction XAS data 11
In situ In reduction XAS data 12
Probing alloying 14
XRD data of bimetallic species 14
Comparative EXAFS data 15
Operando Reaction XAS data 16
Operando XAS mass-spectrometry data 16
Operando Pd reaction XAS data 17
Operando In reaction XAS data 19
Operando DRIFTS data 21
DRIFTS assignments 21
In_2O_3/Al_2O_3 22
Pd/Al_2O_3 24
$Pd/In_2O_3/Al_2O_3$ 26
Supplementary references 28

Experimental protocols

Catalyst synthesis

The Pd-based In_2O_3 and Al_2O_3 catalysts were prepared by co-precipitation followed by calcination. $PdCl_2$ (Sigma Aldrich, 99%) was first dissolved in 10 ml of deionized water with the addition of some drops of HCl (Merck, 37%) under stirring. This solution was added to an $Al(NO_3)_3{\cdot}9H_2O$ and/or $In(NO_3)_3{\cdot}5H_2O$ (Sigma Aldrich) aqueous solution under stirring at room temperature to give a 0.2 M solution with the appropriate cation molar ratios to obtain a final Pd loading of 1 wt.% and an In_2O_3 content of 12 wt.% in the catalyst. A Na_2CO_3 aqueous solution (10 wt.%) was added dropwise to the solution of the metals under stirring at room temperature until a pH of 9.2 was reached. After aging the slurry for 1 h, the precipitate was recovered by filtration, washed with deionized water and dried at 50 °C overnight. The obtained Pd/In/Al hydroxide precursors were calcined at 500 °C for 6 h (heating rate, 10 °C/min). The catalysts were named Pd/Al_2O_3, In_2O_3/Al_2O_3 and $Pd/In_2O_3/Al_2O_3$. For comparison purposes, Pd-In2O3 with 1 Pd wt.%, and In_2O_3 catalysts were prepared following the same procedure as for the mixed oxides, the only difference is that for Pd-In_2O_3 and In_2O_3 the precursors are calcined at 300 °C to prevent excessive loss of active surface.

PdIn/C catalysts were synthesised through the co-decomposition of the metallic precursors $Pd_2(DBA)_3$ (Tris(dibenzylideneacetone)dipalladium(0), Merck, 97%) and $In(acac)_3$ (Indium(III) acetylacetonate, Merck, 99.99%) under H_2 pressure. Specifically, 155.22 mg of $Pd_2(DBA)_3$ and 139.72 mg of $In(acac)_3$ were placed in a Fischer-Porter bottle with 500 mg of activated carbon (Norit D10, Fisher Scientific). Subsequently, 40 mL of mesitylene was added, and the mixture was sonicated for 90 minutes. Then, the Fischer-Porter bottle was pressurized with 3 bar H_2 and heated during 20 h at 180°C. After that, the Fischer-Ported was cooled down, the pressure was released and PdIn/C was filtered through a polyamide membrane (Whatman® membrane filters, 47mmx0.45µm) under vacuum and washed with 50 mL of hexane. Finally, the catalyst was dried overnight in an oven at 60 °C.

Pd_3In/C (15% w/w) was prepared as described for PdIn/C, but adding 237.33 mg of $Pd_2(DBA)_3$ and 71.21 mg of $In(acac)_3$.

Catalyst testing of CO_2 hydrogenation to methanol

The calcined catalysts were pressed at 10 Ton for 20 min to produce a tablet, crushed and sieved to obtain particles with a dimension in the range of 30-40 mesh (0.595-0.400 mm). 0.5 g of the solids were then mixed with SiC (30-40 mesh) to reach a total volume of 1.2 $cm^{3.}$ The sample was placed between two layers of inert material (quartz) in a tubular reactor (INCOLOY 800HT) with an internal diameter of 10 mm and vertically placed into an electric tubular furnace. The gas flow rates were controlled by thermal mass flow controllers (Brooks Instruments), the operating pressure was set by a back-pressure regulator (Swagelok), and all the lines after the reactor outlet were heated at 200 °C to prevent condensation of methanol and water. Before tests, the reduction of the catalysts was performed in situ, flowing a mixture of 25% H_2/N_2 at 120 ml min^{-1}, increasing the oven temperature from 50 to 300 °C (1 °C min^{-1}) and holding this temperature for 2 h. CO_2 hydrogenation to methanol was conducted by feeding a mixture of CO_2 (60% v/v), CH_4 (40% v/v, used as internal standard) and H_2 in a H_2/CO_2 ratio of 3. Catalytic tests were carried out at 20 and 30 bar and GHSV values in the range of 16.800 mL/g_{cat}h. In a typical test, the reactor was purged with N_2 until 175 °C and then pressurized to the working pressure using the reaction mixture. The activity of the catalysts was monitored at an increasing temperature from 175 to 300 °C. The reaction stream was analysed by an online gas chromatograph (Agilent Technology 7890A) equipped with two thermal conductivity detectors (TCD) and a CarboPLOT P7 column using H_2 for the CH_4, CO, CO_2, CH_3OH analyses and an HP-Molesieve column using N_2 for H_2 detection.

High angle annular dark field/scanning tunnelling electron microscopy (HAADF/STEM)

The spent Pd-$12In_2O_3$-Al_2O_3 was investigated with probe corrected FEI-Titan 300keV for HAADF-STEM analysis. The EELS maps were constructed by integrating the Pd M 335 eV, In M 443 eV and O K 532 eV edge after background subtraction.

X-ray photoelectron spectroscopy (XPS)

X-ray photoelectron spectra (XPS) were recorded with a PHI VersaProbe II Scanning XPS Microprobe instrument equipped with a scanning monochromatic X-ray Al Kα radiation as the excitation source (200 µm diameter analysed, 52.8 W, 15kV, 1486.6 eV), and a charge neutralizer; and with PHI5700 instrument with non-monochromatic Al Kα radiation (720 µm diameter analysed, 300 W, 15 kV, 1486,6 eV). The pressure in the analysis chamber was maintained lower than 2.0 x 10^{-6} Pa. High-resolution spectra were recorded at a given take-off angle of 45 ° by a multi-channel hemispherical

electron analyser operating in the constant pass energy mode at 29.35 eV. Spectra were charge referenced with the C 1*s* of adventitious carbon at 284.8 eV. Energy scale was calibrated using Cu $2p_{3/2}$, Ag $3d_{5/2}$, and Au $4f_{7/2}$ photoelectron lines at 932.7, 368.2, and 83.95 eV, respectively. The Multipak software version 9.6.0.15 was employed to analyse in detail the recorded spectra.[1] The obtained spectra were fitted using Gaussian–Lorentzian curves to more accurately extract the binding energies of the different element core levels. The catalysts were reduced ex-situ under a hydrogen flow (H_2/N_2 1/3 GHSV= 14 400 ml/h·g cat) in a tubular reactor by heating over a linear temperature ramp (1°C/min) from room temperature to 300 °C, remaining for 2h at that temperature. After reduction, the samples were cooled to room temperature under a He flow and kept in cyclohexane in sealed vials to prevent oxidation. Before XPS analyses, samples were transferred on a sample holder in a dry box under N_2 atmosphere, and then were transferred in a special transference system into the introduction chamber of the XPS system and were subjected to high vacuum overnight to remove cyclohexane and other physically adsorbed species prior to their introduction in the analysis chamber.

Operando X-ray absorption spectroscopy (XAS)

X-ray absorption spectroscopy was performed at Diamond Light Source, UK, at B18, through proposal SP30647 for the Pd/Al_2O_3, Pd/TiO_2 and $Pd/In_2O_3/Al_2O_3$ species. Data was collected at the Pd and In K-edges in transmission mode using a QEXAFS setup with fast scanning Si(311) crystal monochromators. The time resolution here was roughly 90 seconds per spectrum. Samples were packed into an in-house custom-made capillary system designed and constructed by Diamond Light Source. 50 mg of 200-300 µm catalyst particles were packed between 3 cm of quartz wool (Figure S1).

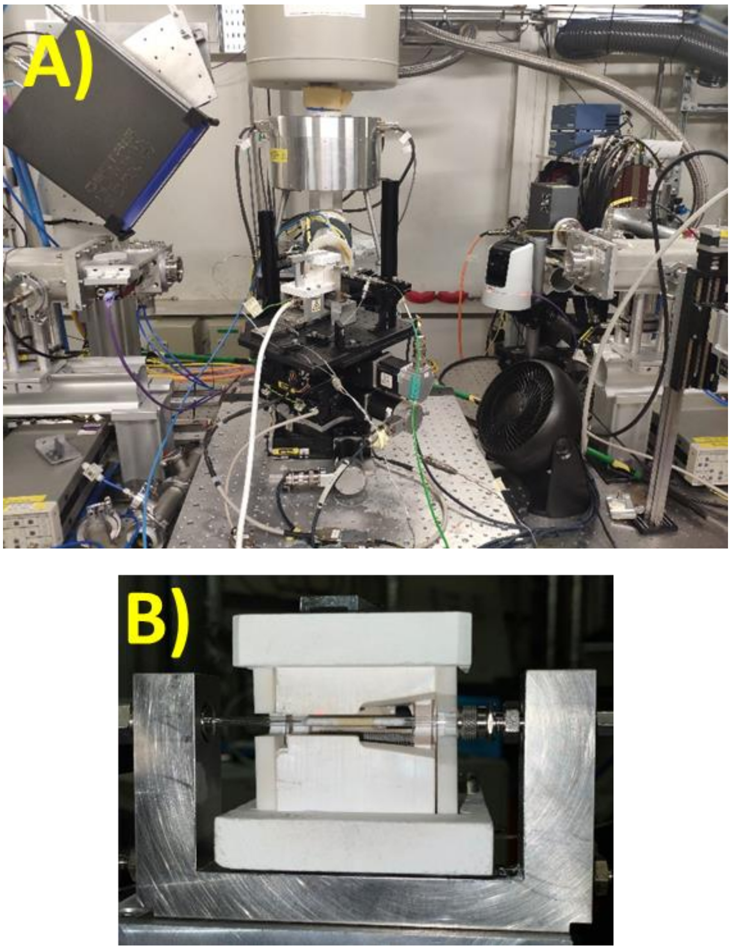

Figure S1: A) Beamline set-up and B) Pressurised microreactor used for the *operando* XAS measurements on B18.

For the reduction step, the sample was subjected to a flow of 30 mL/min of 4% H_2 in He, at atmospheric pressure. The system was heated from 30 to 300 °C at a ramp rate of 5 °C/min and a dwell time of 1 hour. XAS data was constantly collected throughout this process, with Pd and In measurements alternated. The system was then cooled down to 30 °C.

For the reaction step, the sample was first pressurised to 20 bar at 30 °C using 47 mL/min of 4% H_2 in He and 2.5 mL/min of 25% CO_2 in He to give a 3:1 molar ratio of H_2:CO_2. Once pressurised, the temperature was ramped at 10 °C/min, pausing at 50, 100, 150, 200 and 250 °C for 30 minutes to collect spectra.

Mass spectrometry data was collected throughout using a Pfeiffer OmniStar spectrometer, with particular focus on the following species:

Table S1: Mass-spectrometry signals of interest for *operando* CO_2 hydrogenation to methanol

m/z	Species	m/z	Species
2	H_2	28	CO
4	He	29	H_2CO/CH_3OH
14	N_2	31	CH_3OH
16	O_2 & CH_4	32	O_2
18	H_2O	44	CO_2
27	CH_3OH	46	HCOOH

Data was analysed and fit using the Strawberry Demeter packages Athena and Artemis.[2] Due to a difference in data quality arising from the different loadings of In and Pd in the bimetallic sample, different fitting processes were used.

For Pd, the amplitude factor (S_0^2) was determined to be 0.775 from fitting the Pd foil reference. Pd spectra were then fit using a Hanning window function in a 3.0 to 11.0 k-range and a 1.0 to 2.8 R-range, and fitting exclusively to a k^3 weighting. Pd-O (~ 2.0 Å) and Pd-Pd (~ 2.7 Å) paths were both considered, with the most accurate combination of Pd-O and/or Pd-Pd presented herein. Attempts were made to include third and fourth terms for the cumulant expansion for the Debye Waller factor, however this led to a shortening of the Pd-Pd values to an unrealistic value of 2.61 Å.

For In, the amplitude factor was found to be 0.937 from fitting the In foil reference. In spectra were also fitted using a Hanning window function in a 3.0 to 11.0 k-range and a 1.0 to 3.0 R-range. Data were simultaneously fit to k^1, k^2 and k^3 weightings. Only In-O paths (~ 2.1 Å) are reported, as the addition of other paths (In-In at 3.2 Å, In-Pd at 2.7 to 2.8 Å etc.) did not lead to improved fitting in any case.

Data collected on the PdIn/C and Pd_3In/C, after *in situ* reduction, collected under identical conditi0ons to Diamond Light Source, were collected on beamline BM31 at the European Synchrotron Radiation Facility (ESRF).

Operando diffuse reflectance infrared Fourier transform spectroscopy (DRIFTS)

DRIFTS were performed on a Bruker VERTEX 80 system, with a custom-built high-pressure dome, capable of holding 25 bar of pressure within a high pressure chamber DiffusIR^{TM} PIKE Technologies accessory. Argon and a 25% CO_2/75% H_2 mixture gas were delivered through high pressure EL-FLOW® Bronkhorst mass-flow controllers, into an in-house custom-made gas manifold.

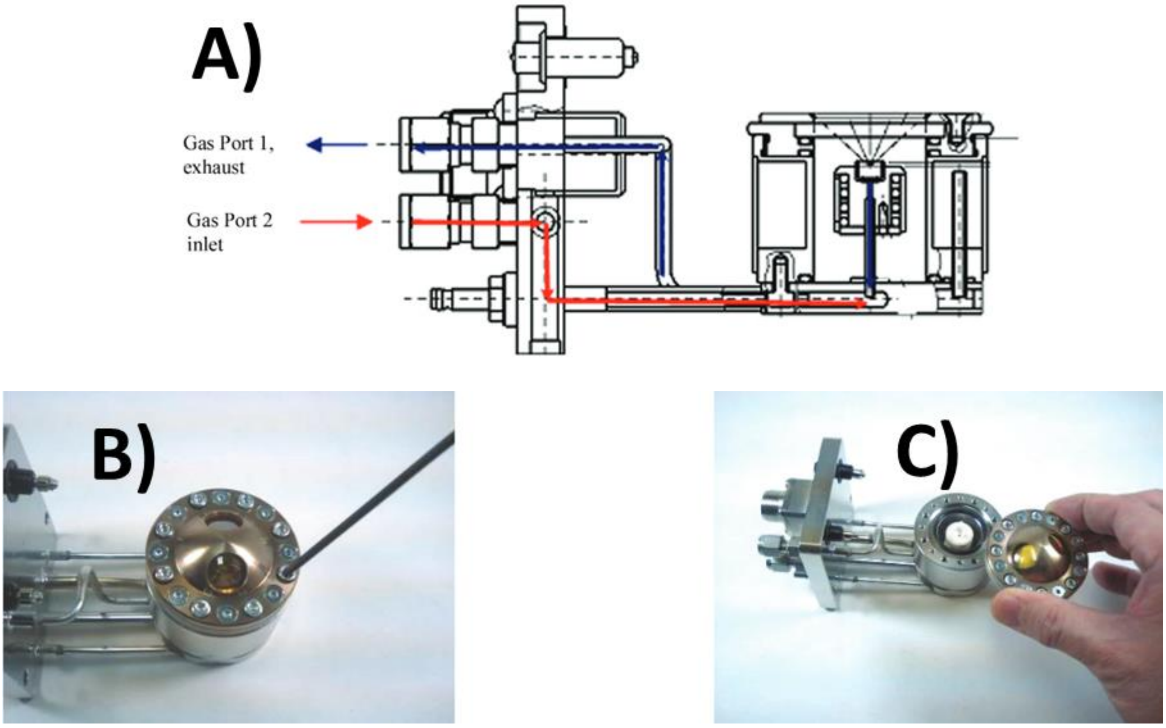

Figure S2: A) Schematic and B) & C) Pictures showing the high pressure DRIFTS dome used for *operando* DRIFTS measurements

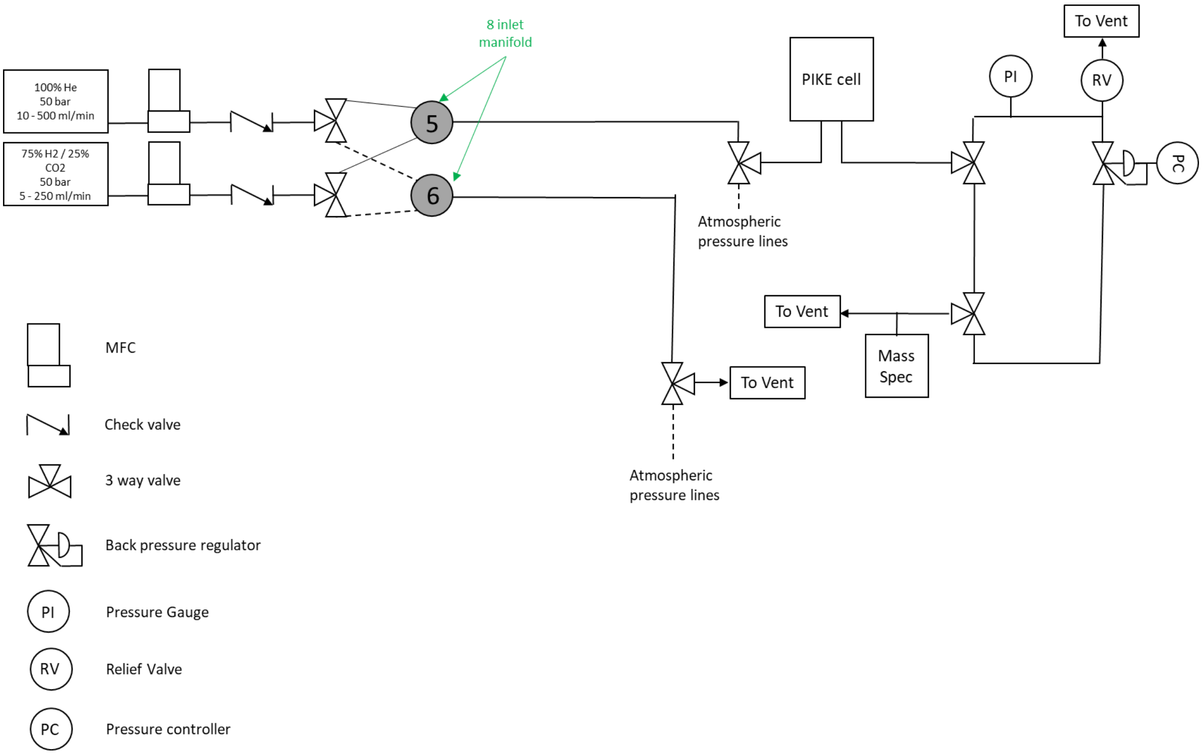

Figure S3: Schematic of the in-house custom-made gas delivery system for *operando* DRIFTS measurements.

30 mg of sample was used for the measurements. Initially, the sample was reduced in 25 mL/min of 25% H_2 and 75 % Ar. The system was heated to 300 °C at a ramp rate of 5 °C/min and held for 1 hour. The samples were then cooled to 30 °C and either pressurised to 20 bar with 30 mL/min of 75% H_2 and 25 % CO_2 for the 20 bar measurements or kept at atmospheric pressure for the 1 bar measurements. Once the intended final pressure was reached, the system was heated at 10 °C/min, while holding at 50, 100, 150, 175, 200, 225, 250, 275 and 300 °C for 30 minutes for spectra to be collected. Mass-spectrometry data was collected throughout to observe the evolution of different species, using a Pfeiffer OmniStar.

A polished mirror was used as a background. Reported data are difference spectra, with the 30 °C reaction spectra subtracted to show the evolution of the FTIR signals with temperature. The two regions, C-H and carbonate are presented by subtracting a linear background over the 3150 and 2650 cm^{-1} range, so that the intensity at 3150 and 2650 cm^{-1} are both 0. An identical process was performed over the 2100 and 1200 cm^{-1} range also.

Catalytic data

CO_2 hydrogenation to methanol

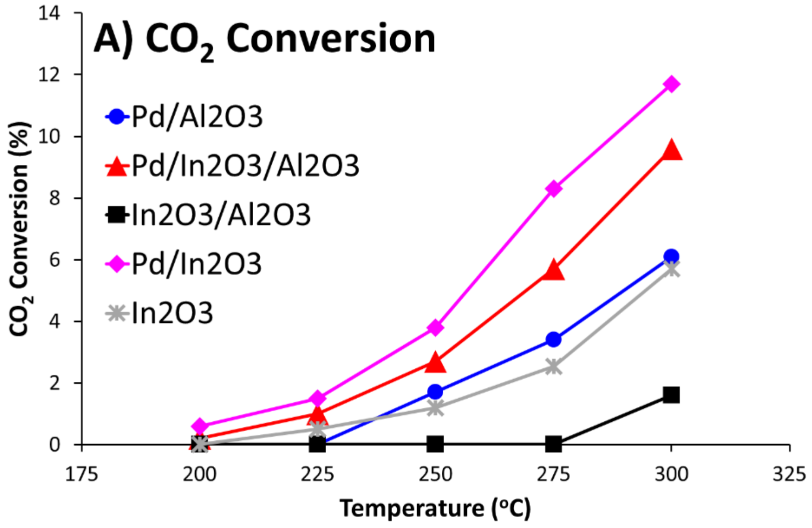

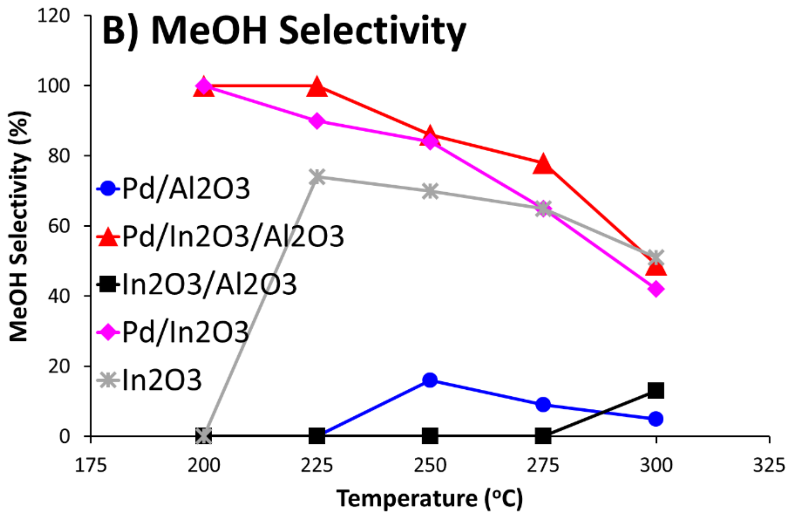
B) MeOH Selectivity
MeOH Selectivity (%)
Pd/Al2O3
Pd/In2O3/Al2O3
In2O3/Al2O3
Pd/In2O3
In2O3
Temperature (°C)

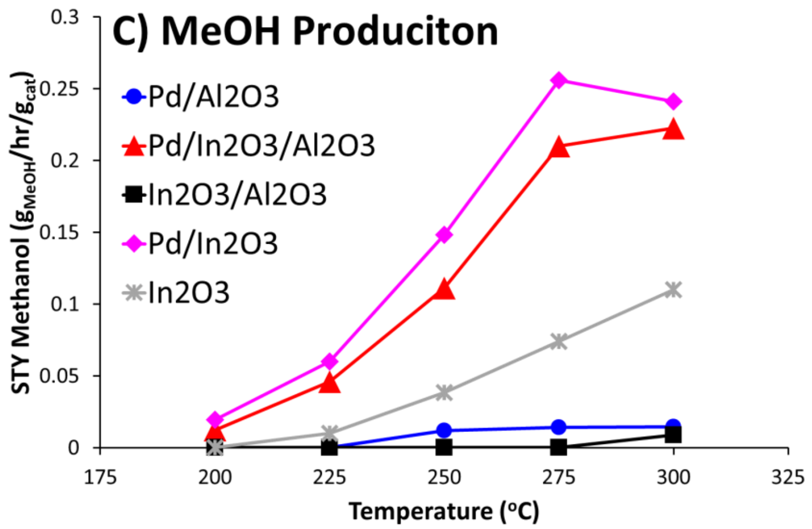
C) MeOH Produciton
STY Methanol (g_{MeOH}/hr/g_{cat})
Pd/Al2O3
Pd/In2O3/Al2O3
In2O3/Al2O3
Pd/In2O3
In2O3
Temperature (°C)

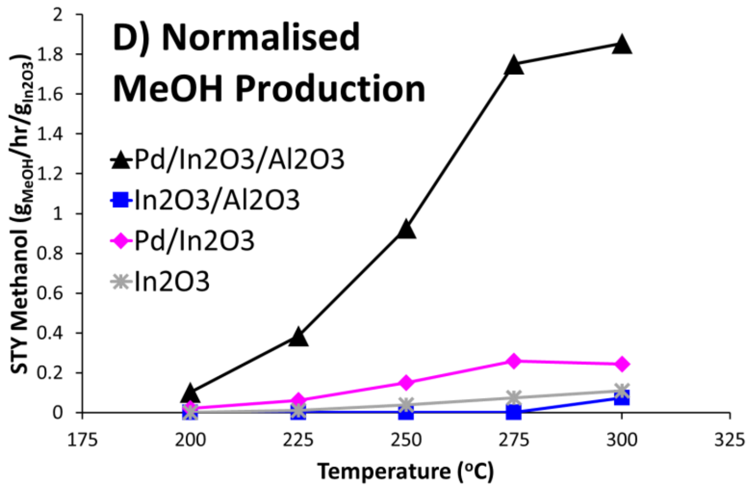
D) Normalised MeOH Production
STY Methanol (g_{MeOH}/hr/g_{In2O3})
Pd/In2O3/Al2O3
In2O3/Al2O3
Pd/In2O3
In2O3
Temperature (°C)

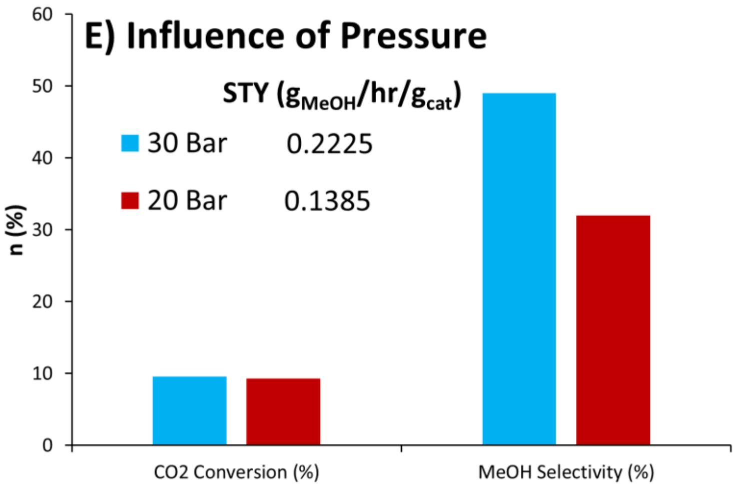

Figure S4: Catalytic CO_2 hydrogenation to methanol data, for testing parameters see experimental methods section, highlighting A) Total CO_2 conversion, B) MeOH selectivity, C) Total methanol production and D) Methanol production normalised by In_2O_3 quantity at 30 bar of pressure. E) Shows the comparative performance at 20 bar compared to 30 bar. Despite having a fraction of the Pd/In_2O_3 mass, the Pd/In_2O_3/Al_2O_3 system shows similar activity to the pure Pd/In_2O_3 system, and has greatly improved activity with regards to In_2O_3 content.

Ex situ characterisation

High angle annular dark field scanning tunnelling electron microscopy (STEM-HAADF)

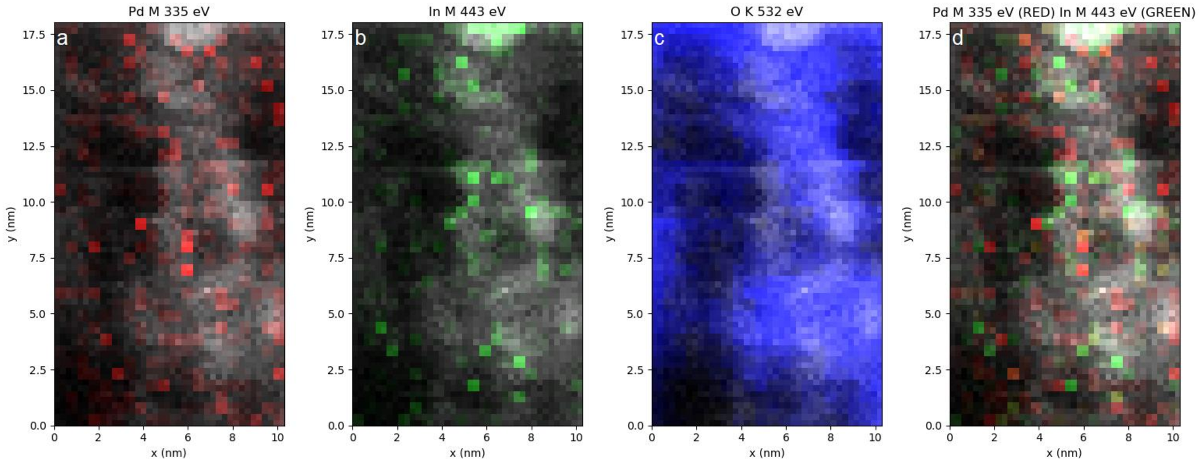

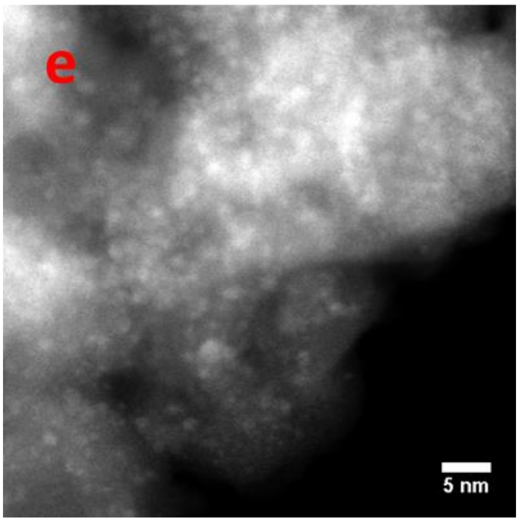

Figure S5: STEM-EELS maps of the spent $Pd/In_2O_3/Al_2O_3$. The Pd M, In M and O K edge overlaid on top of the HAADF-STEM acquired at the same time (a-c). The Pd M (Red) and In M (green) edge mapped together. Both signals represented in the same pixel as yellow, and e) STEM-HAADF image of the spent $Pd/In_2O_3/Al_2O_3$. Based on 11 images of this sample the mean size is found to be 1.3nm ± 0.3nm based on 195 particles measured.

X-ray photoelectron spectroscopy (XPS)

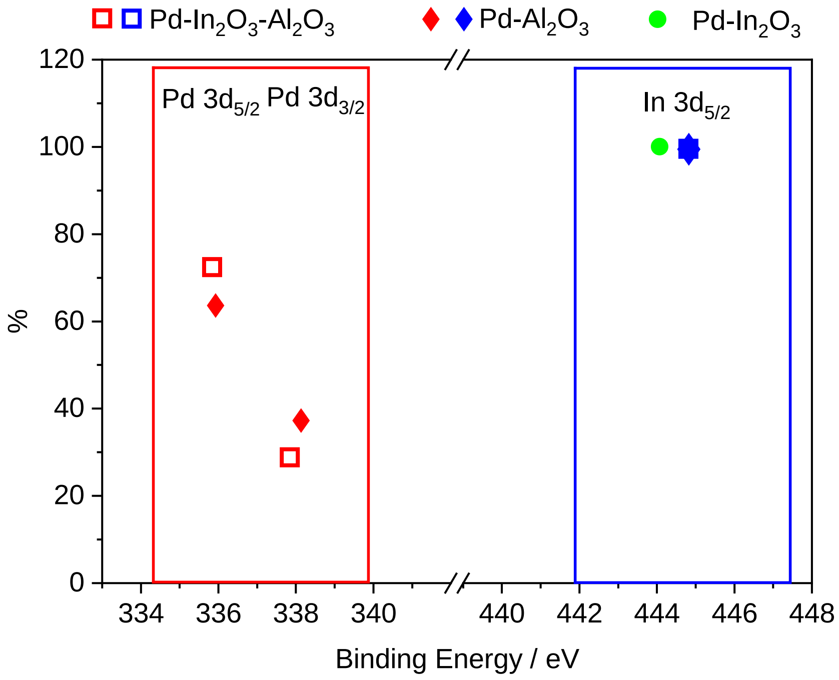

Figure S6: Pd $3d_{5/2}$, Pd $3d_{3/2}$ and In $3d_{5/2}$ binding energy values of calcined catalysts.

In situ Reduction XAS data

In situ Pd reduction XAS data

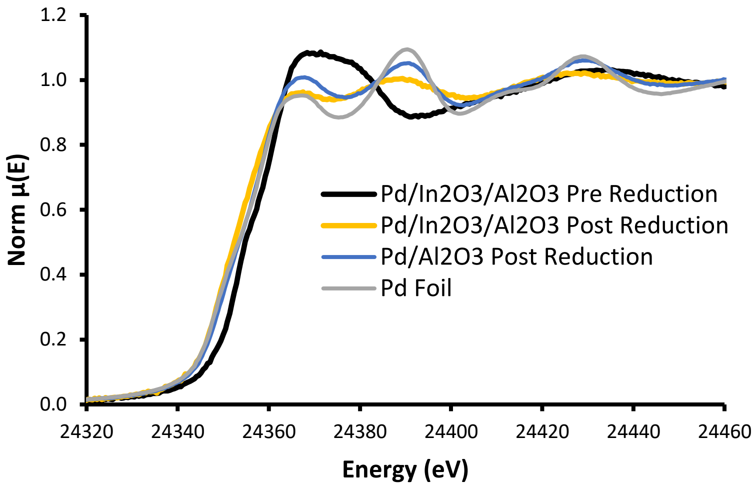

Figure S7: Comparing the XANES position of Pd/In_2O_3/Al_2O_3 before and after reduction, showing a significant shift towards Pd foil and reduced Pd/Al_2O_3, confirming the reduction of the system.

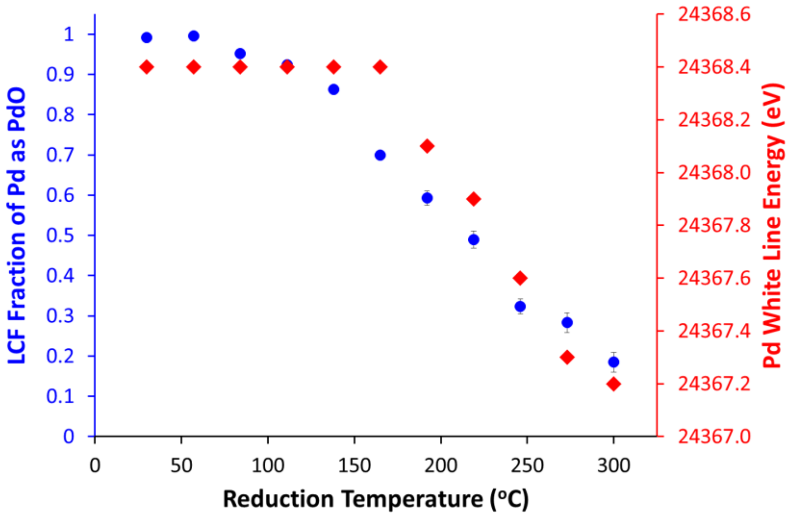

Figure S8: Pd K-edge XANES analysis Pd/In_2O_3/Al_2O_3 showing the trend in the decrease in the fractional composition of PdO through linear combination fitting analysis, and the change in the Pd white line energy, as a function of increasing reduction temperature from 30 to 300 °C. LCF fitting was performed using a 2-component fit of PdO and metallic Pd, with the 30 °C spectra serving as the PdO reference, and the Pd foil as the metallic Pd reference; we note that these references cannot fully capture the subtle and inevitable deviations in the spectra due to the Pd species in the sample being nano-sized, however they do allow us to illustrate the reduction in Pd oxidation state. This is also borne out by a strong correlation between this increasing extent of reduction and the shift in white line energy.

In situ In reduction XAS data

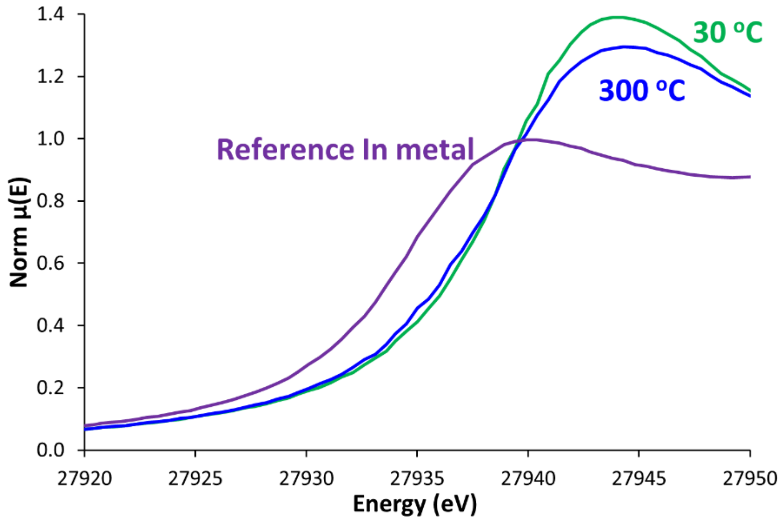

Figure S9: In K-edge XANES focussing of Pd/In_2O_3/Al_2O_3 on the pre-edge signal, showing the subtle decrease in pre-edge energy on increasing the reduction temperature from 30 °C to 300 °C, relative to the metallic In reference foil.

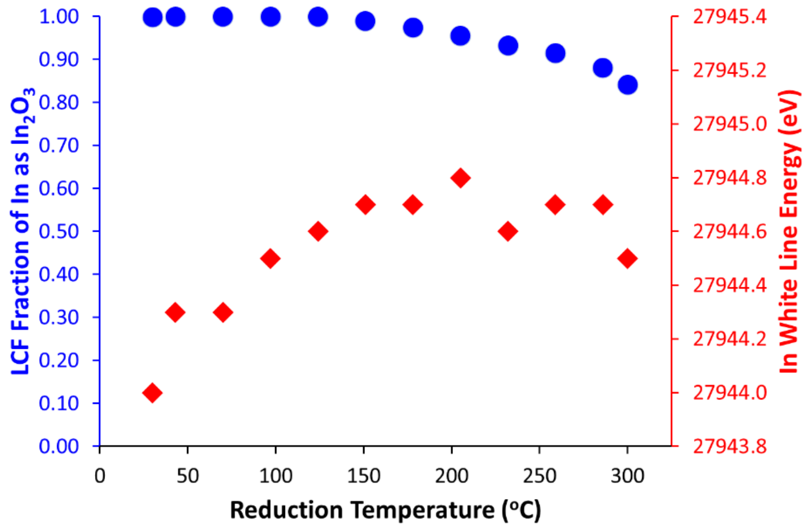

Figure S10: In K-edge XANES analysis of Pd/In_2O_3/Al_2O_3 during reduction showing a slight decrease in the fractional composition of In_2O_3 through linear combination fitting analysis, and no real deviation in white line energy, as a function of increasing reduction temperature from 30 to 300 °C. LCF fitting was performed using a 2-component fit of In_2O_3 and metallic In, with the 30 °C spectra serving as the In_2O_3 reference, and the In foil as the metallic In reference. This suggests there is no significant reduction of In_2O_3 to In metal occurring.

Table S2: Fitted EXAFS parameters for the In K-edge EXAFS data during reduction from 30 to 300 °C.

			Path 1 In-O		
System	R-factor	deltaE	n1	r1	σ_1^2

30 °C	0.0081	4.4 ± 1.0	5.82 ± 0.49	2.13 ± 0.01	0.007 ± 0.001
43 °C	0.0076	4.4 ± 0.9	5.74 ± 0.47	2.13 ± 0.01	0.008 ± 0.001
70 °C	0.0079	4.4 ± 1.0	5.73 ± 0.49	2.13 ± 0.01	0.008 ± 0.001
97 °C	0.0080	4.5 ± 1.0	5.71 ± 0.49	2.12 ± 0.01	0.008 ± 0.001
124 °C	0.0074	4.5 ± 0.9	5.60 ± 0.46	2.12 ± 0.01	0.008 ± 0.001
151 °C	0.0082	4.2 ± 1.0	5.49 ± 0.46	2.12 ± 0.01	0.008 ± 0.001
178 °C	0.0098	4.0 ± 1.0	5.31 ± 0.48	2.12 ± 0.01	0.008 ± 0.001
205 °C	0.0091	3.9 ± 1.0	5.18 ± 0.47	2.11 ± 0.01	0.008 ± 0.001
232 °C	0.0095	4.1 ± 1.1	4.93 ± 0.45	2.11 ± 0.01	0.008 ± 0.001
259 °C	0.0133	3.4 ± 1.0	4.78 ± 0.39	2.10 ± 0.01	0.008 ± 0.001
286 °C	0.0127	3.4 ± 1.2	4.51 ± 0.46	2.10 ± 0.01	0.008 ± 0.002
300 °C	0.0143	3.2 ± 1.2	4.26 ± 0.45	2.10 ±0 .01	0.008 ± 0.002
30 °C Post*	0.0171	3.2 ± 1.3	3.90 ± 0.43	2.10 ± 0.01	0.007 ± 0.002

*"30 °C Post" refers to data collected after reduction (post-reduction), still under a hydrogen atmosphere, but collected at 30 °C. As this was collected at the same temperature as the "30 °C" data, then a direct comparison can be made between the two species, with the influence of thermal effects ignored. Fitted using a Hanning Window, in a 3.0 to 11.0 k-range and a 1.0 to 3.0 R-range, and fitted simultaneously to k^1, k^2 and k^3 weightings. The amplitude factor (S_0^2) was determined to be 0.937 from fitting the In foil reference. Only In-O paths (~ 2.1 Å) are reported, as the addition of other paths (In-In at 3.2 Å, In-Pd at 2.7 to 2.8 Å etc.) did not lead to improved fitting in any case.

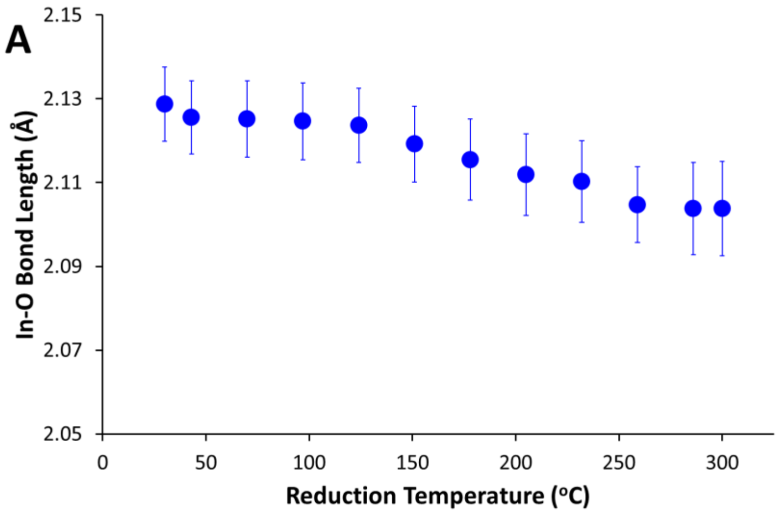

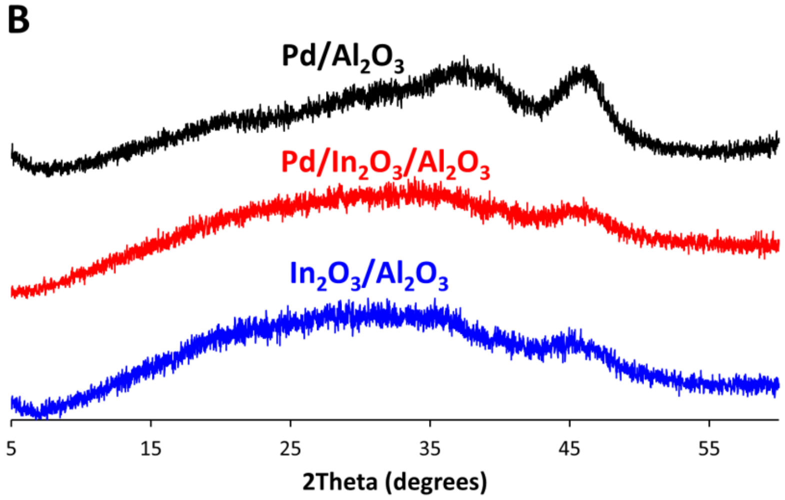

Figure S11: A) In K-edge EXAFS fitting parameters for $Pd/In_2O_3/Al_2O_3$ during reduction, showing the evolution of the In-O bond length, with calculated errors, as a function of increasing reduction temperature from 30 to 300 °C. The initial value at 30 °C (2.13 ± 0.01) spans the range of 2.14 to 2.12 Å, whilst the final value at 300 °C (2.10 ± 0.01), spans the range 2.11 to 2.09 Å, which is outside of error, suggesting a modest shortening. B) Powder XRD data showing the lack of long-range order in the Pd, In_2O_3 and Al_2O_3, showing signals due to γ-Al_2O_3 but not bixbyite In_2O_3.

Probing alloying

XRD data of bimetallic species

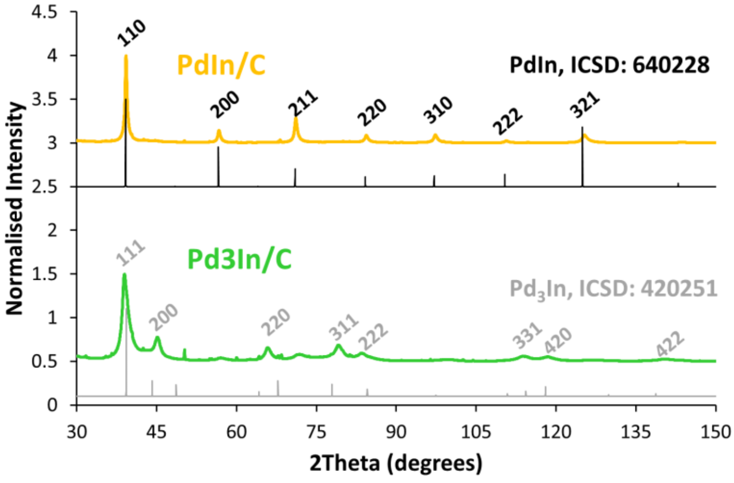

Figure S12: Powder XRD patterns of PdIn/C and Pd_3In/C. The PdIn/C (yellow) shows exclusively signals due to the PdIn alloy (black), whereas most signals in Pd_3In/C are attributed to the Pd_3In alloy. In both cases the appearance of crystalline PdIn phases shows significant alloying has occurred. Indexing performed by matching the peaks to simulated XRD patterns, taken from cif files from references [3] (**PdIn**) and [4] (**Pd_3In**).

Comparative EXAFS data

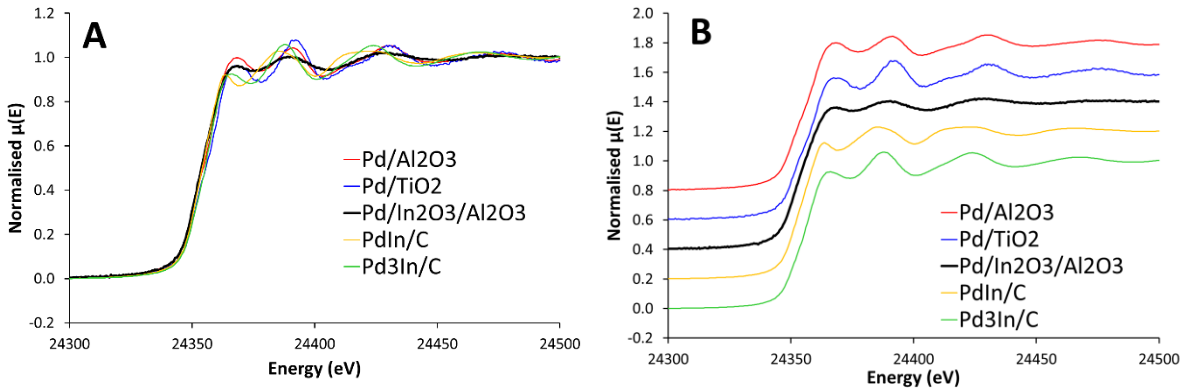

Figure S13: Comparative XANES data showing data A) Overlaid and B) Stacked by an increment of 0.2 in μ(E). The positions of the first two peaks in the EXAFS region of the $Pd/In_2O_3/Al_2O_3$ species, and their relative intensities better match the monometallic Pd/Al_2O_3 species than the bimetallic alloys.

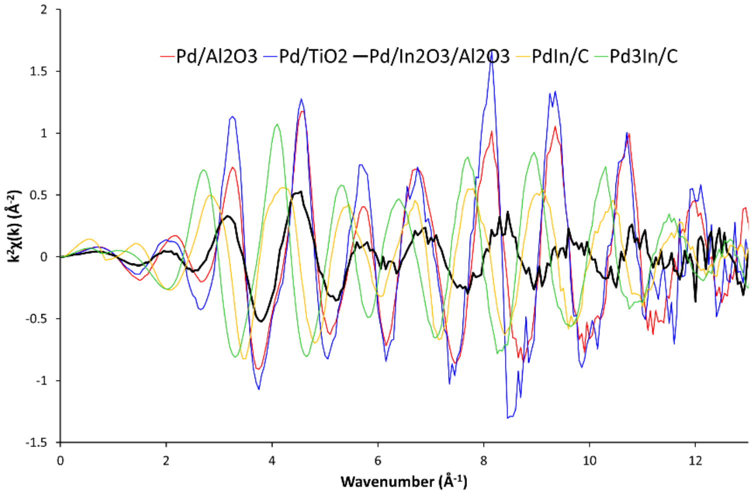

Figure S14: Comparative EXAFS data showing the k-plot data A) Overlaid and B) Stacked by an increment of 0.7 in $k^2\chi(k)$. The oscillations of the $Pd/In_2O_3/Al_2O_3$ closely resemble the monometallic Pd/Al_2O_3 and Pd/TiO_2 species, however, differs noticeably from the bimetallic species; PdIn/C and Pd_3In/C, suggesting the $Pd/In_2O_3/Al_2O_3$ species better resembles the monometallic species.

Table S3: EXAFS fitting of model monometallic and bimetallic species, post-reduction, collected at 30 ˚C under hydrogen atmosphere.

			Path 1 Pd-O			Path 2 Pd-M		
**System**	**R**	**Enot**	**n1**	**r1**	**ss1**	**n2**	**r2**	**ss2**
Pd/Al_2O_3	0.018	2.4 ± 1.3				8.59 ± 1.46	2.73 ± 0.01	0.007 ± 0.001
Pd/TiO_2	0.005	2.6 ± 0.7				11.54 ± 1.00	2.74 ± 0.01	0.007 ± 0.001
**Pd/In_2O_3 /Al_2O_3**	**0.038**	**-3.3 ± 3.0**	**1.32 ± 1.65**	**2.00 ± 0.05**	**0.010 ± 0.014**	**4.86 ± 1.92**	**2.64 ± 0.02**	**0.013 ± 0.003**
**Pd/In_2O_3 /Al_2O_3**	**0.110**	**-4.5 ± 2.9**				**5.67 ± 2.13**	**2.64 ± 0.02**	**0.014 ± 0.003**
PdIn/C	0.029	-4.1 ± 1.6				7.82 ± 1.61	2.78 ± 0.01	0.008 ± 0.001
Pd_3In/C	0.005	-3.0 ± 0.6				9.37 ± 0.74	2.78 ± 0.01	0.008 ± 0.001

Fitted using a Hanning Window, in a 3.0 to 11.0 k-range and a 1.0 to 2.8 R-range, and fitted exclusively to a k^3 weighting. The amplitude factor (S_0^2) was determined to be 0.775 from fitting the Pd foil reference. Pd-O (~ 2.0 Å) and Pd-In/Pd (Pd-M) (~ 2.7 Å) paths were both considered, with the most accurate combination of Pd-O and/or Pd-Pd presented herein. Two fits were considered for the $Pd/In_2O_3/Al_2O_3$, one including just a Pd-M path, and one with a Pd-O and a Pd-M path. In both cases the Pd-M distance remained at 2.64 ± 0.02 Å.

Operando Reaction XAS data

Operando XAS mass-spectrometry data

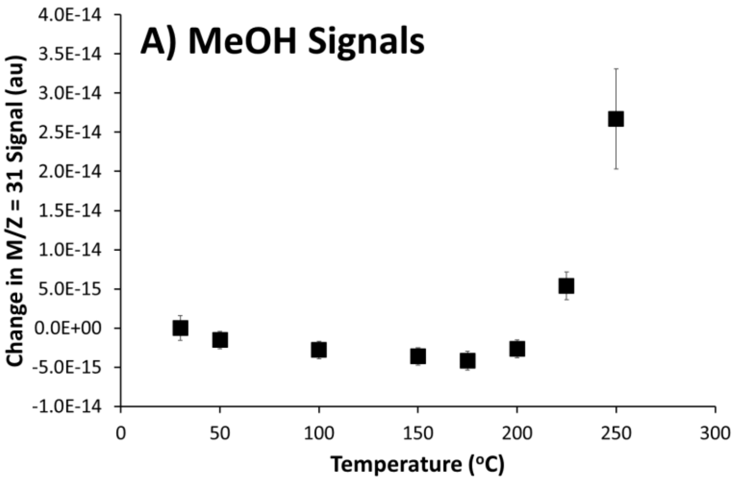

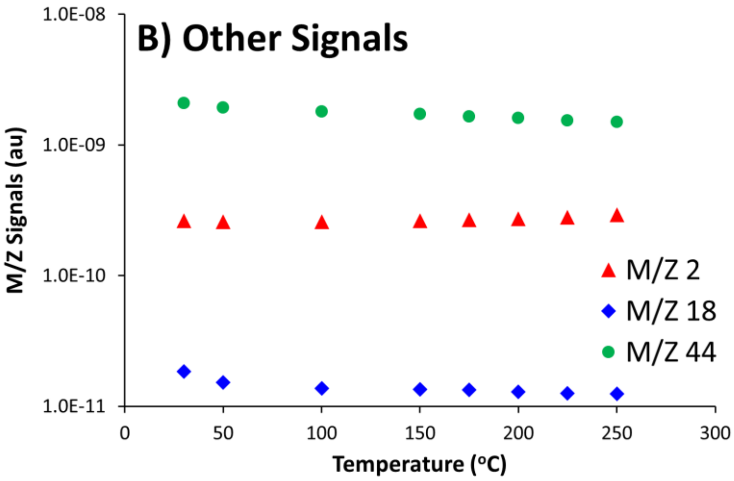

Figure S15: Mass-spectrometry data simultaneously collected during the *operando* XAS data, focussing on A) Methanol; m/z = 31, and B) m/z = 2, 18 and 44 (H_2, H_2O and CO_2). Data points for each temperature are the average of 100 points, collected over the final 15-minutes of the 30-minute hold period. Errors presented are the calculated standard deviations for those 100 points. The data is presented so that the value at 30 °C is the 0 point. The m/z = 31 signal (MeOH) clearly grows with temperature, beyond the margins of calculated error.

Operando Pd reaction XAS data

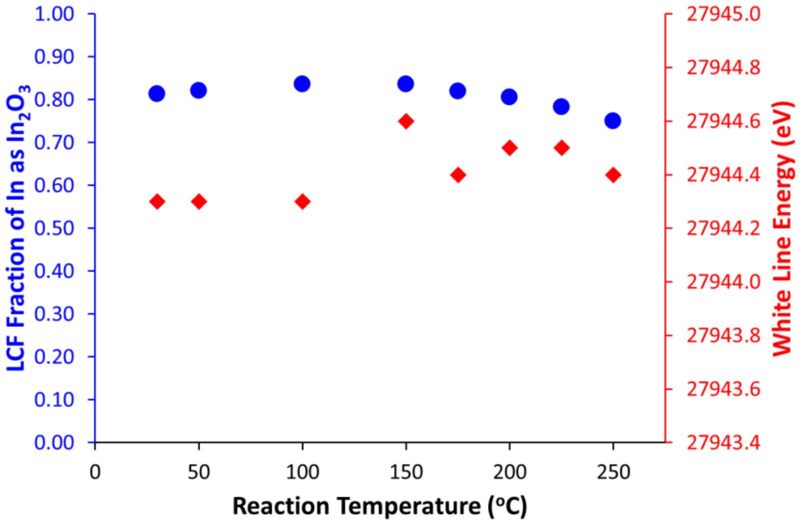

Figure S16: Pd K-edge XANES analysis of $Pd/In_2O_3/Al_2O_3$ during the CO_2 hydrogenation to methanol reaction, showing no change in PdO fractional composition through linear combination fitting analysis, and no real deviation in white line energy, as a function of increasing reduction temperature from 30 to 250 °C. This suggests there is no significant change in Pd environment occurring during the reaction.

Table S4: Fitted EXAFS parameters for the Pd K-edge EXAFS data during reaction.

			Path 1 Pd-O			Path 2 Pd-Pd		
**System**	**R-factor**	**deltaE**	**n1**	**r1**	σ_1^2	**n2**	**r2**	σ_2^2
30 °C	0.0297	-4.5 ± 2.5	1.66 ± 1.68	2.01 ± 0.04	0.013 ± 0.013	5.67 ± 1.86	2.65 ± 0.02	0.016 ± 0.003
50 °C	0.0166	-4.0 ± 1.9	1.00 ± 0.71	2.00 ± 0.03	0.007 ± 0.007	5.37 ± 1.33	2.65 ± 0.01	0.014 ± 0.002
100 °C	0.0259	-4.2 ± 2.3	1.13 ± 1.03	1.99 ± 0.04	0.008 ± 0.009	5.44 ± 1.69	2.63 ± 0.02	0.015 ± 0.003
150 °C	0.0155	-4.4 ± 1.8	1.02 ± 0.83	1.99 ± 0.03	0.008 ± 0.008	5.51 ± 1.32	2.63 ± 0.01	0.014 ± 0.002
175 °C	0.0214	-4.1 ± 2.1	1.25 ± 1.24	2.00 ± 0.04	0.011 ± 0.011	5.31 ± 1.49	2.63 ± 0.01	0.014 ± 0.002
200 °C	0.0154	-4.6 ± 1.8	1.12 ± 0.94	2.00 ± 0.03	0.010 ± 0.009	5.37 ± 1.27	2.62 ± 0.01	0.014 ± 0.002
225 °C	0.0211	-5.1 ± 2.1	0.75 ± 0.58	1.99 ± 0.03	0.003 ± 0.006	5.39 ± 1.53	2.62 ± 0.01	0.015 ± 0.002
250 °C	0.0236	-4.5 ± 2.5	1.17 ± 1.61	1.98 ± 0.06	0.012 ± 0.017	**4.42** ± 1.39	2.62 ± 0.01	0.012 ± 0.002

Fitted using a Hanning Window, in a 3.0 to 11.0 k-range and a 1.0 to 2.8 R-range, and fitted exclusively to a k^3 weighting. The amplitude factor (S_0^2) was determined to be 0.775 from fitting the Pd foil reference. Pd-O (~ 2.0 Å) and Pd-Pd (~ 2.7 Å) paths were both considered, with the most accurate combination of Pd-O and/or Pd-Pd presented herein. The "Path 2 Pd-Pd" CN value at 250 °C (4.42 ± 1.39) is believed to be due to an artifact of the fitting process, given the significant difference from all other values, and the similarity of the XANES, EXAFS and R-space plots.

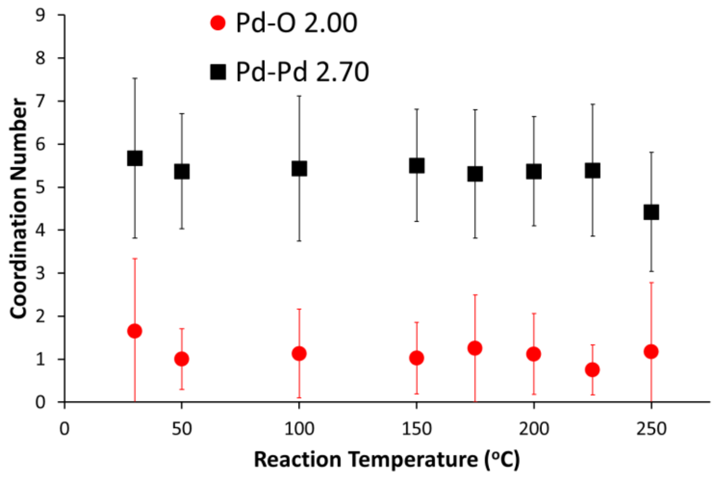

Figure S17: Pd K-edge EXAFS fitting parameters for $Pd/In_2O_3/Al_2O_3$ during the CO_2 hydrogenation to methanol reaction, showing no significant change in Pd-Pd and Pd-O coordination number, presented with calculated errors, as a function of increasing reaction temperature.

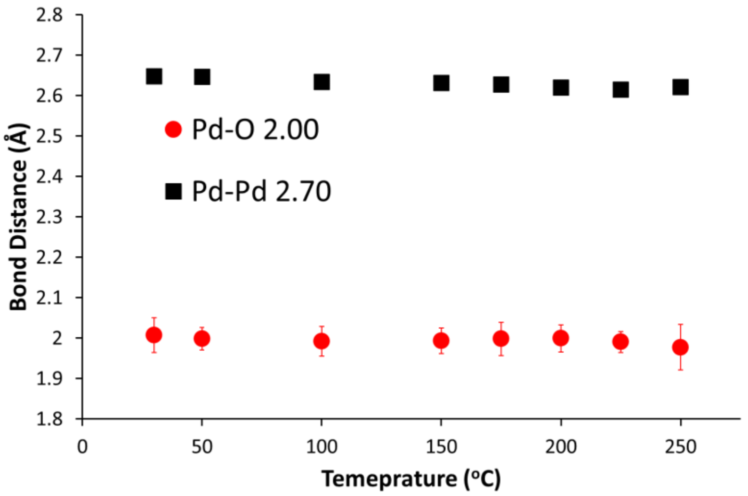

Figure S18: Pd K-edge EXAFS fitting parameters for $Pd/In_2O_3/Al_2O_3$ during the CO_2 hydrogenation to methanol reaction, showing consistent Pd-Pd and Pd-O bond lengths, with calculated errors, as a function of increasing reaction temperature from 30 to 250 °C. The Pd-O and Pd-Pd bond lengths show little variation.

Operando In reaction XAS data

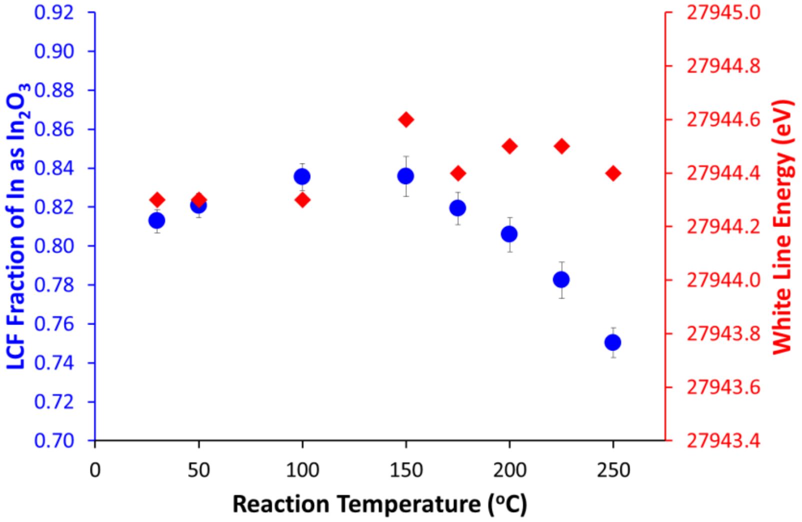

Figure S19: In K-edge XANES analysis of $Pd/In_2O_3/Al_2O_3$ during the CO_2 hydrogenation to methanol reaction, showing only subtle changes in the In_2O_3 fractional composition through linear combination fitting analysis, and no real deviation in white line energy, as a function of increasing reduction temperature from 30 to 250 °C. This suggests there is no significant change in In environment occurring during the reaction.

Table S5: Fitted EXAFS parameters for the In K-edge EXAFS data during reaction.

			Path 1 In-O		
**System**	**R-factor**	**deltaE**	**n1**	**r1**	**σ_1^2**
30 °C	0.0118	3.1 ± 1.1	4.34 ± 0.42	2.13 ± 0.01	0.007 ± 0.001
50 °C	0.0127	3.6 ± 1.2	4.31 ± 0.43	2.13 ± 0.01	0.008 ± 0.001
100 °C	0.0135	3.7 ± 1.2	4.42 ± 0.46	2.13 ± 0.01	0.008 ± 0.001
150 °C	0.0138	3.7 ± 1.2	4.33 ± 0.46	2.12 ± 0.01	0.008 ± 0.001
175 °C	0.0138	3.9 ± 1.3	4.29 ± 0.46	2.12 ± 0.01	0.008 ± 0.001
200 °C	0.0141	3.5 ± 1.2	4.24 ± 0.45	2.12 ± 0.01	0.008 ± 0.001
225 °C	0.0143	3.4 ± 1.2	4.14 ± 0.44	2.12 ± 0.01	0.008 ± 0.001
250 °C	0.0134	3.5 ± 1.2	3.96 ± 0.42	2.11 ± 0.01	0.008 ± 0.001

Fitted using a Hanning Window, in a 3.0 to 11.0 k-range and a 1.0 to 3.0 R-range, and fitted simultaneously to k^1, k^2 and k^3 weightings. The amplitude factor (S_0^2) was determined to be 0.937 from fitting the In foil reference. Only In-O paths (~ 2.1 Å) are reported, as the addition of other paths (In-In at 3.2 Å, In-Pd at 2.7 to 2.8 Å etc.) did not lead to improved fitting in any case.

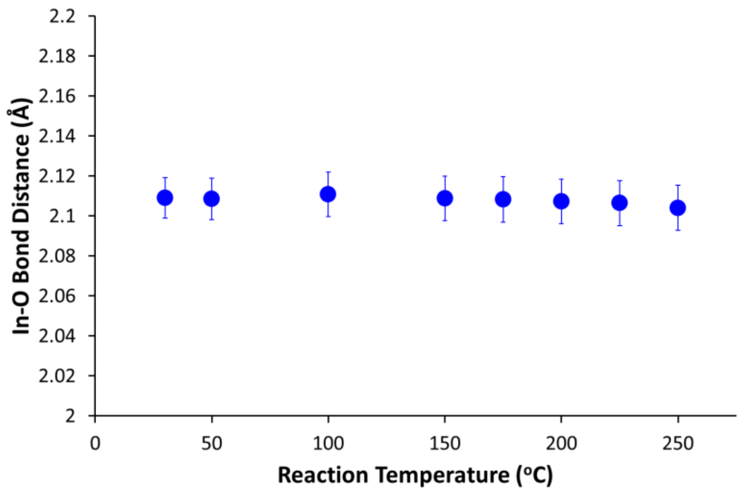

Figure S20: In K-edge EXAFS fitting parameters for $Pd/In_2O_3/Al_2O_3$ during the CO_2 hydrogenation to methanol reaction, showing no significant change in In-O bond distance, presented with calculated errors, as a function of increasing reaction temperature from 30 to 250 °C.

Operando DRIFTS data

DRIFTS assignments

Table S6: DRIFTS assignments with literature values

Experimental Values / (cm^{-1}) In_2O_3/Al_2O_3	Pd/Al_2O_3	$Pd/In_2O_3/Al_2O_3$	Assignment	Literature Value / (cm^{-1})	Ref
		**C-H Region (3150 – 2650 cm^{-1})**			
3015	3016	3016	Methane	3016<br>3016	[5]<br>[6]
2998	2996	--	Formate	2997<br>2996<br>2996	[7]<br>[8]<br>[9]
2960	2948	2950	Methanol	2952<br>2940	[6]<br>[10]
2923	2925	--	Methoxy	2928<br>2927<br>2920	[11]<br>[12]<br>[13]
2902	2906	2906	Formate	2902<br>2902<br>2900	[8]<br>[7]<br>[9]
--	2888	2881	Formate	2888<br>2886<br>2878	[8]<br>[14]<br>[15]
--	--	2866	Formate	2874<br>2870<br>2867	[14]<br>[9]<br>[16]
2852	2846	2842	Methoxy	2850<br>2849<br>2843	[17]<br>[18]<br>[7]
2742	2742	2742	Formate	2742<br>2737<br>2735	[7]<br>[14]<br>[15]
		**Carbonate Region (2100 – 1200 cm^{-1})**			
--	2030	2040	Linear CO	2050<br>2038	[5]<br>[15]
--	1890	--	CO	1882	[8]
1672	1666	1672	Bidentate carbonate	1680<br>1672<br>1668	[11]<br>[19]<br>[20]
1606	1606	1606	Formate	1609<br>1609<br>1604	[6]<br>[13]<br>[8]
1517	1517	1517	Unidentate carbonate	1520	[21]

1392	1392	1396	Formate	1393<br>1388	[5]<br>[14]
1323	1315	1323	Polydentate carbonate	1327<br>1323	[7]<br>[6]
1305	--	1305	Formate	1294	[13]

In_2O_3/Al_2O_3

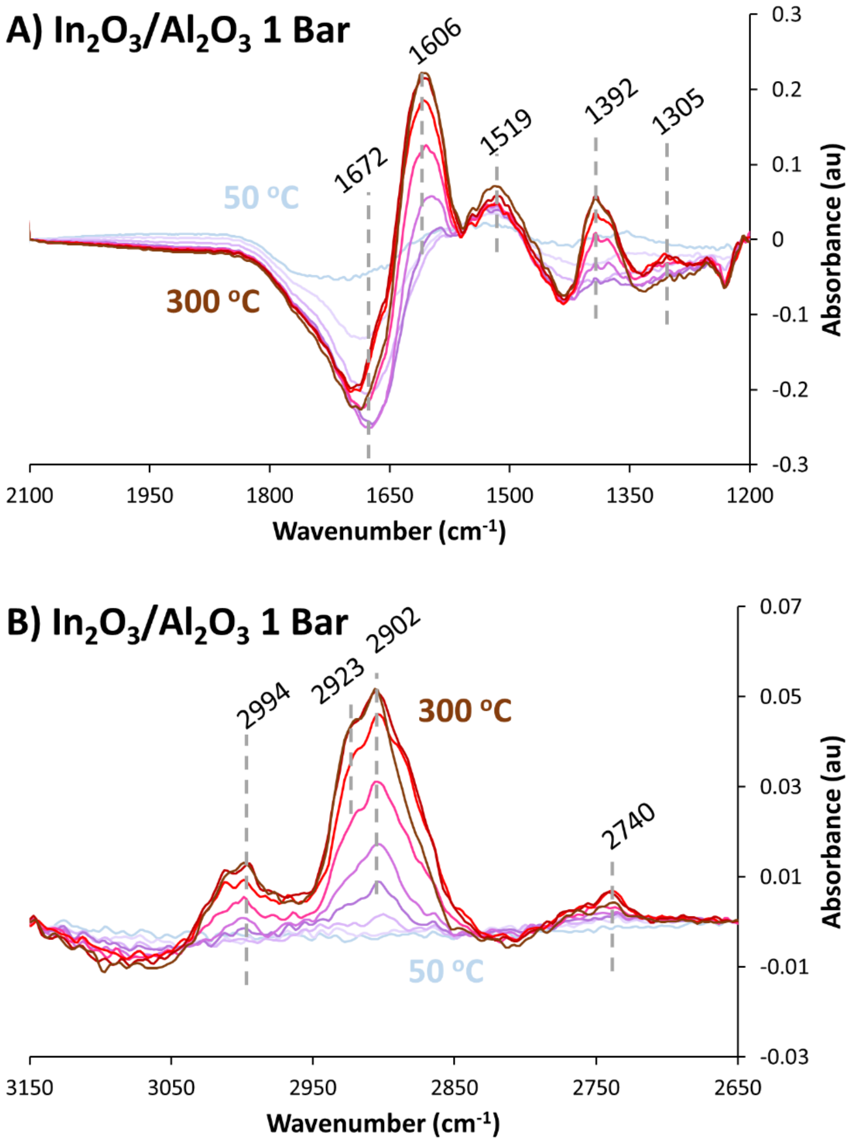

Figure S21: *Operando* DRIFTS difference data of In_2O_3/Al_2O_3 collected from 50 to 300 °C. The spectra at 30 °C was subtracted from all temperatures (50, 100, 150, 175, 200, 225, 250, 275 and 300 °C), spectra were then normalised between A) 2100 – 1200 cm^{-1}, to emphasise the carbonate region and B) 3150 – 2650 cm^{-1} to show the C-H region. Data was collected under 1 bar of pressure, with 30 mL/min of 75% H_2 and 25% CO_2 (3:1 molar ratio). All data was collected after 30 minutes of equilibration. Unlike the XAS data, DRIFTS data were collected up to 300 °C to emphasise the change in signals for ease of assignment.

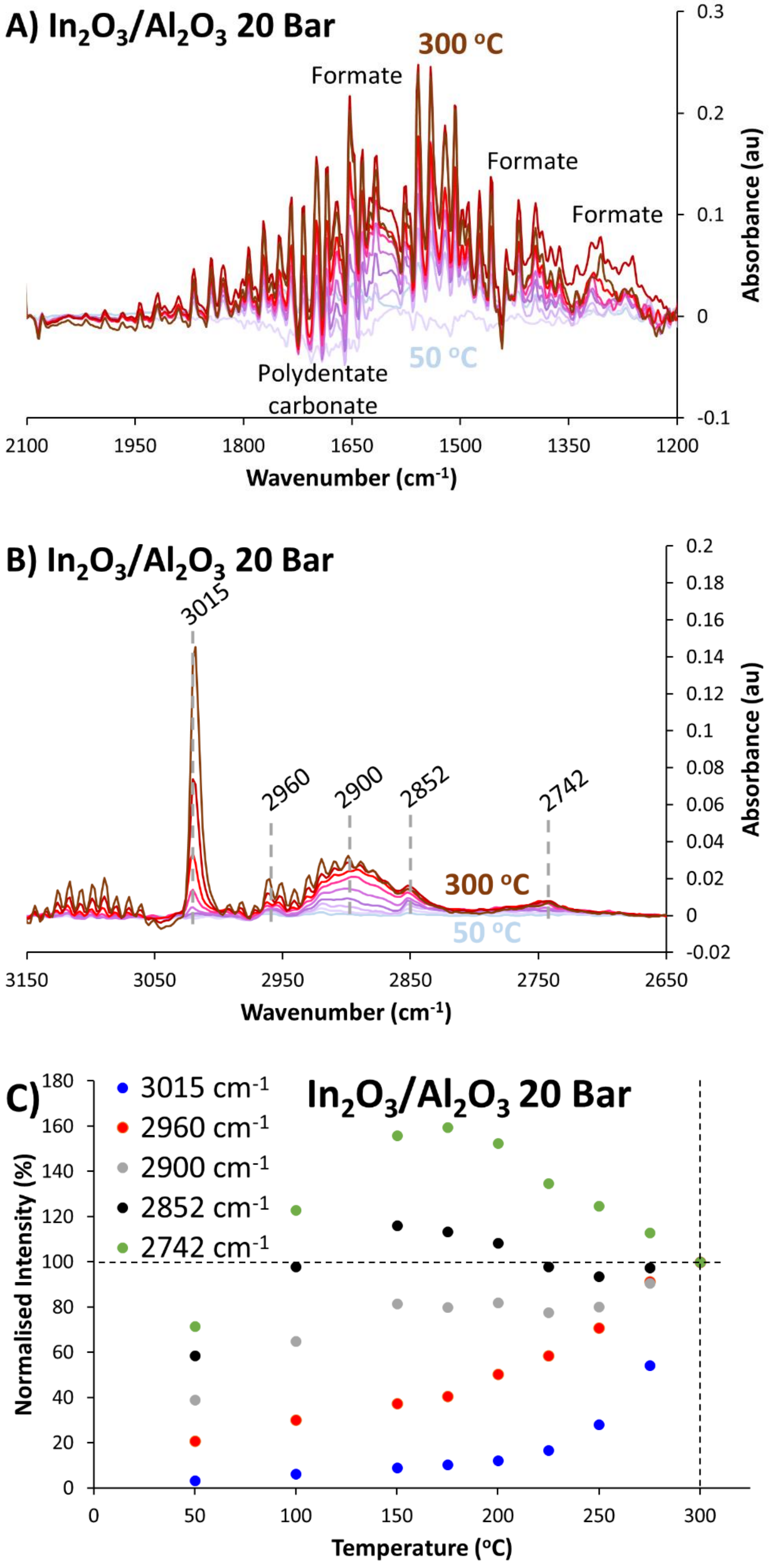

Figure S22: *Operando* DRIFTS difference data of In_2O_3/Al_2O_3 collected from 50 to 300 °C. The spectra at 30 °C was subtracted from all temperatures (50, 100, 150, 175, 200, 225, 250, 275 and 300 °C),

spectra were then normalised between A) 2100 – 1200 cm^{-1}, to emphasise the carbonate region and B) 3150 – 2650 cm^{-1} to show the C-H region. Data was collected under 20 bar of pressure, with 30 mL/min of 75% H_2 and 25% CO_2 (3:1 molar ratio). All data was collected after 30 minutes of equilibration. Unlike the XAS data, DRIFTS data were collected up to 300 °C to emphasise the change in signals for ease of assignment. C) Showing the progression of signal intensities as a function of temperature, normalised at 300 °C.

Pd/Al_2O_3

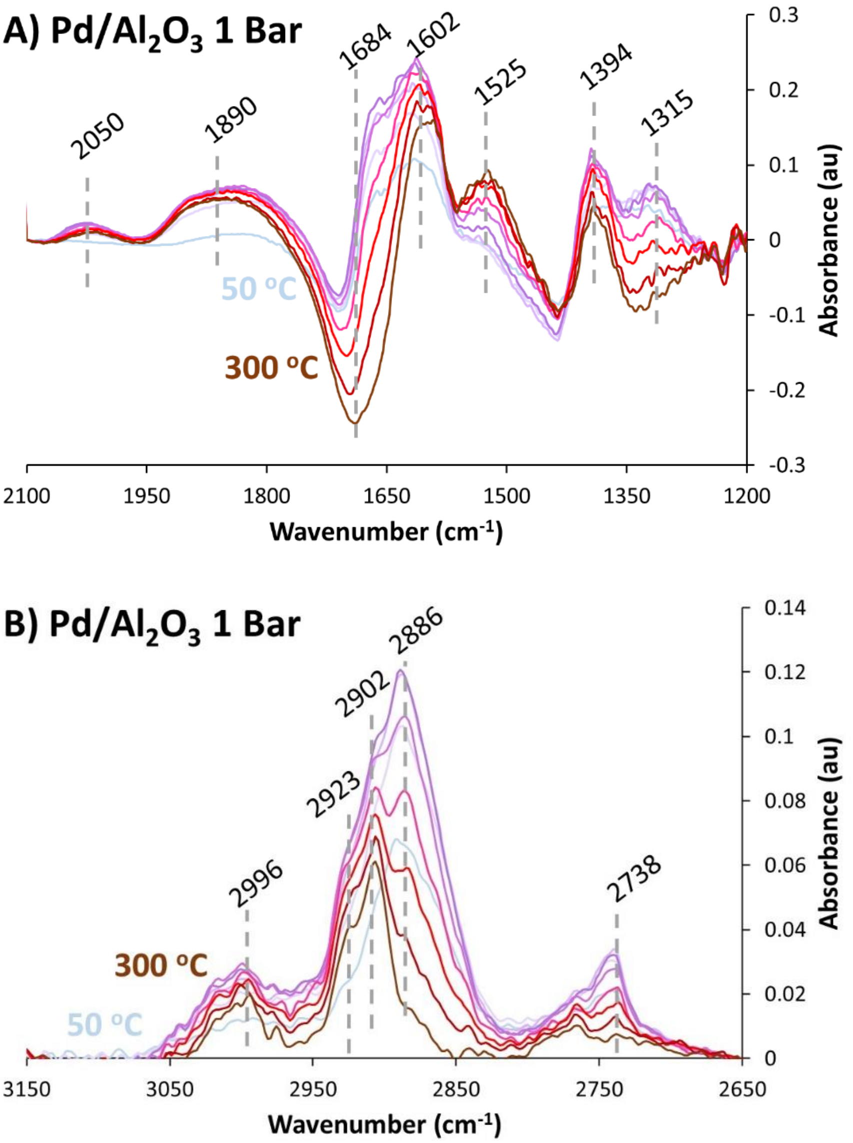

Figure S23: *Operando* DRIFTS difference data of Pd/Al_2O_3 collected from 50 to 300 °C. The spectra at 30 °C was subtracted from all temperatures (50, 100, 150, 175, 200, 225, 250, 275 and 300 °C), spectra were then normalised between A) 2100 – 1200 cm^{-1}, to emphasise the carbonate region and B) 3150 – 2650 cm^{-1} to show the C-H region. Data was collected under 1 bar of pressure, with 30 mL/min of 75% H_2 and 25% CO_2 (3:1 molar ratio). All data was collected after 30 minutes of equilibration. Unlike the XAS data, DRIFTS data were collected up to 300 °C to emphasise the change in signals for ease of assignment.

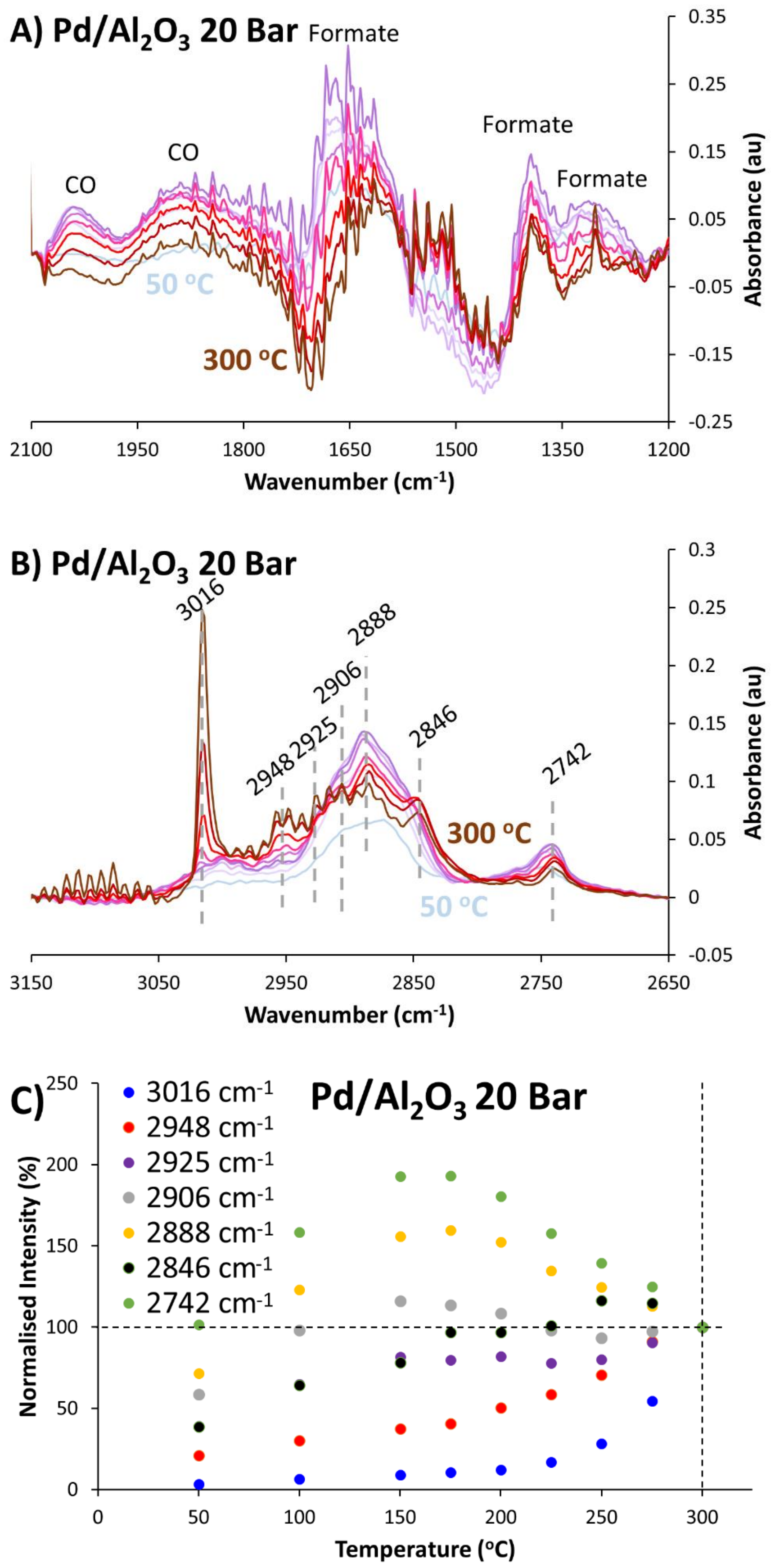
A) Pd/Al2O3 20 Bar
Formate
Formate
Formate
CO
CO
50 oC
300 oC
Absorbance (au)
Wavenumber (cm-1)
B) Pd/Al2O3 20 Bar
3016
2948
2925
2906
2888
2846
2742
300 oC
50 oC
Absorbance (au)
Wavenumber (cm-1)
C) Pd/Al2O3 20 Bar
3016 cm-1
2948 cm-1
2925 cm-1
2906 cm-1
2888 cm-1
2846 cm-1
2742 cm-1
Normalised Intensity (%)
Temperature (oC)

Figure S24: *Operando* DRIFTS difference data of Pd/Al_2O_3 collected from 50 to 300 °C. The spectra at 30 °C was subtracted from all temperatures (50, 100, 150, 175, 200, 225, 250, 275 and 300 °C), spectra were then normalised between A) 2100 – 1200 cm^{-1}, to emphasise the carbonate region and B) 3150 – 2650 cm^{-1} to show the C-H region. Data was collected under 20 bar of pressure, with 30 mL/min of 75% H_2 and 25% CO_2 (3:1 molar ratio). All data was collected after 30 minutes of equilibration. Unlike the XAS data, DRIFTS data were collected up to 300 °C to emphasise the change in signals for ease of assignment. C) Showing the progression of signal intensities as a function of temperature, normalised at 300 °C.

Pd/In_2O_3/Al_2O_3

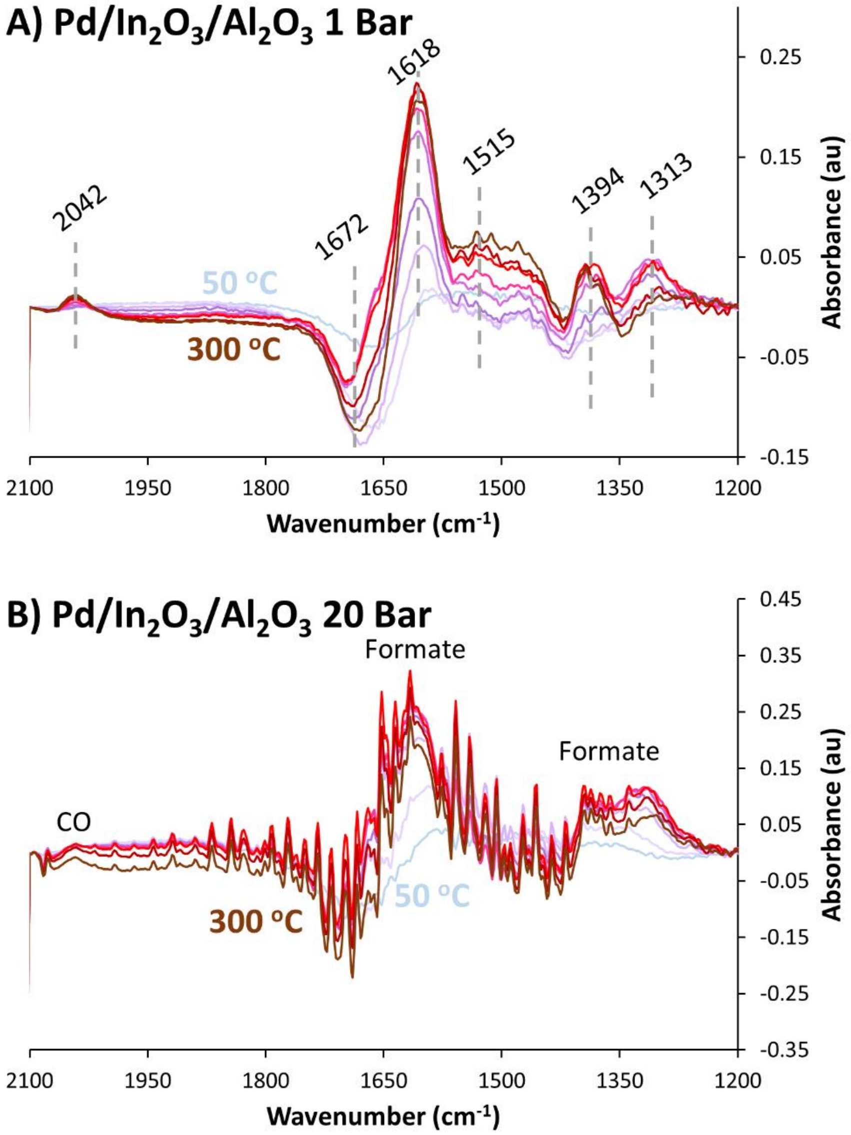

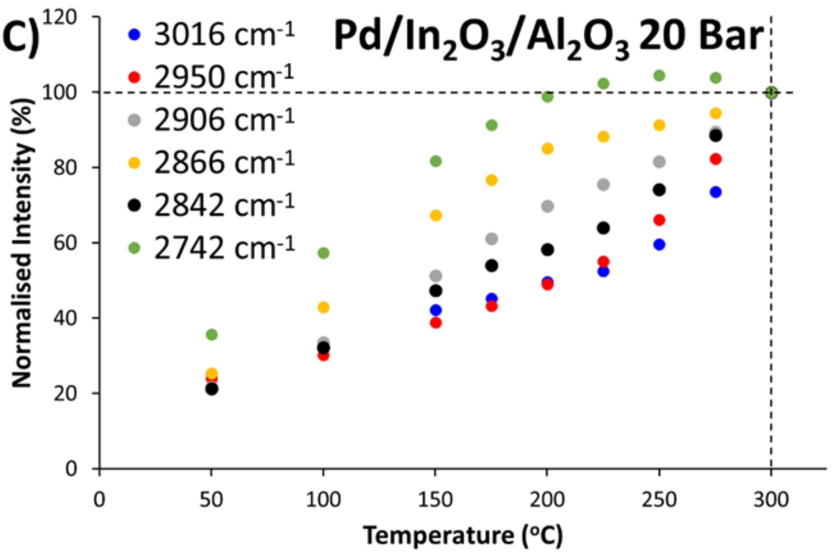

Figure S25: *Operando* DRIFTS difference data of Pd/In_2O_3/Al_2O_3 collected from 50 to 300 °C. The spectra at 30 °C was subtracted from all temperatures (50, 100, 150, 175, 200, 225, 250, 275 and 300 °C), spectra were then normalised between 2100 – 1200 cm^{-1}, to emphasise the carbonate region. Data was collected under A) 1 bar and B) 20 bar of pressure, with 30 mL/min of 75% H_2 and 25% CO_2 (3:1 molar ratio). All data was collected after 30 minutes of equilibration. Unlike the XAS data, DRIFTS data were collected up to 300 °C to emphasise the change in signals for ease of assignment. C) Showing the progression of signal intensities as a function of temperature, normalised at 300 °C.

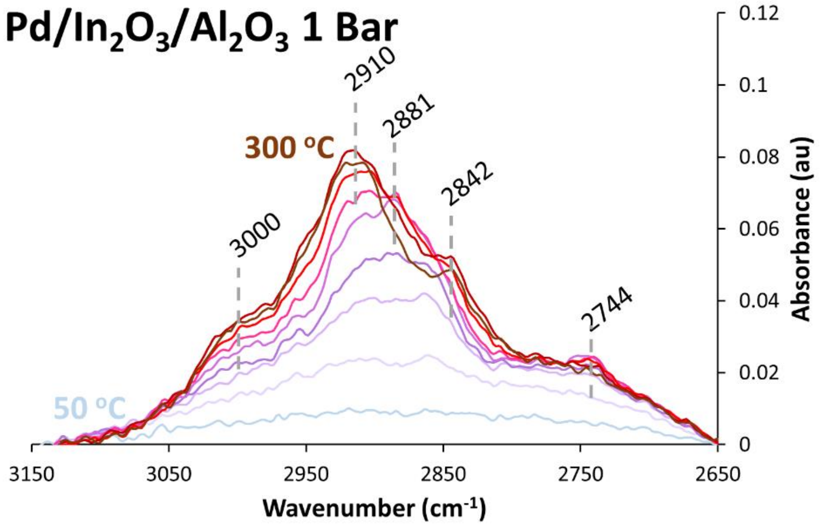

Figure S26: *Operando* DRIFTS difference data of Pd/In_2O_3/Al_2O_3 collected from 50 to 300 °C. The spectra at 30 °C was subtracted from all temperatures (50, 100, 150, 175, 200, 225, 250, 275 and 300 °C), spectra were then normalised between 3150 – 2650 cm^{-1}, to emphasise the C-H region. Data was collected under 1 bar of pressure, with 30 mL/min of 75% H_2 and 25% CO_2 (3:1 molar ratio). All data was collected after 30 minutes of equilibration. Unlike the XAS data, DRIFTS data were collected up to 300 °C to emphasise the change in signals for ease of assignment.

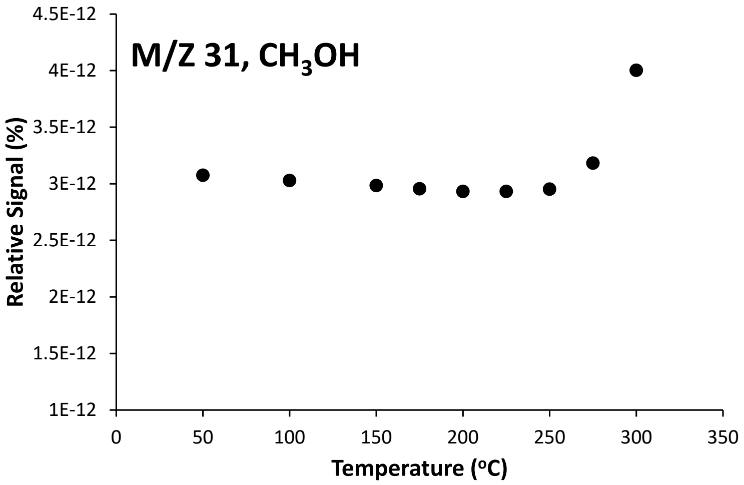

Figure S27: Mass-spectrometry data for $Pd/In_2O_3/Al_2O_3$ for the *operando* DRIFTS experiments, showing the formation of methanol as a function of reaction temperature.

Note: copies of the XAFS and IR data can be found via the following doi: 10.5522/04/22240831

Supplementary references

[1] https://www.phi.com/surface-analysis-equipment/system-software/multipak-data-reduction-software.html.

[2] B. Ravel, M. Newville, *J Synchrotron Radiat* **2005**, *12*, 537-541.

[3] I. R. Harris, M. Norman, A. W. Bryant, *Journal of the Less Common Metals* **1968**, *16*, 427-440.

[4] H. Kohlmann, C. Ritter, *Zeitschrift für anorganische und allgemeine Chemie* **2009**, *635*, 1573-1579.

[5] X. Wang, H. Shi, J. H. Kwak, J. Szanyi, *ACS Catalysis* **2015**, *5*, 6337-6349.

[6] R. Khobragade, M. Roškarič, G. Žerjav, M. Košiček, J. Zavašnik, N. Van de Velde, I. Jerman, N. N. Tušar, A. Pintar, *Applied Catalysis A: General* **2021**, *627*.

[7] L. Wang, U. J. Etim, C. Zhang, L. Amirav, Z. Zhong, *Nanomaterials (Basel)* **2022**, *12*.

[8] A. Kaftan, M. Kusche, M. Laurin, P. Wasserscheid, J. Libuda, *Applied Catalysis B: Environmental* **2017**, *201*, 169-181.

[9] L. F. Bobadilla, J. L. Santos, S. Ivanova, J. A. Odriozola, A. Urakawa, *ACS Catalysis* **2018**, *8*, 7455-7467.

[10] S. M. Fehr, K. Nguyen, I. Krossing, *ChemCatChem* **2021**, *14*.

[11] C. Y. Regalado Vera, N. Manavi, Z. Zhou, L.-C. Wang, W. Diao, S. Karakalos, B. Liu, K. J. Stowers, M. Zhou, H. Luo, D. Ding, *Chemical Engineering Journal* **2021**, *426*.

[12] C. Huang, Z. Wu, H. Luo, S. Zhang, Z. Shao, H. Wang, Y. Sun, *ACS Applied Energy Materials* **2021**, *4*, 9258-9266.

[13] L. Yao, X. Shen, Y. Pan, Z. Peng, *J Catal* **2019**, *372*, 74-85.

[14] S. Kattel, B. Yan, Y. Yang, J. G. Chen, P. Liu, *J Am Chem Soc* **2016**, *138*, 12440-12450.

[15] K. Lee, U. Anjum, T. P. Araújo, C. Mondelli, Q. He, S. Furukawa, J. Pérez-Ramírez, S. M. Kozlov, N. Yan, *Applied Catalysis B: Environmental* **2022**, *304*.

[16] W. Wang, Z. Qu, L. Song, Q. Fu, *J Catal* **2020**, *382*, 129-140.

[17] T. Shi, Y. Men, S. Liu, J. Wang, Z. Li, K. Qin, D. Tian, W. An, X. Pan, L. Li, *Colloids and Surfaces A: Physicochemical and Engineering Aspects* **2022**, *651*.

[18] S. Dang, B. Qin, Y. Yang, H. Wang, J. Cai, Y. Han, S. Li, P. Gao, Y. Sun, *Sci Adv* **2020**, *6*, eaaz2060.

[19] T. Das, G. Deo, *Journal of Molecular Catalysis A: Chemical* **2011**, *350*, 75-82.

[20] S. Natesakhawat, P. R. Ohodnicki, B. H. Howard, J. W. Lekse, J. P. Baltrus, C. Matranga, *Topics in Catalysis* **2013**, *56*, 1752-1763.
[21] M. S. Frei, C. Mondelli, A. Cesarini, F. Krumeich, R. Hauert, J. A. Stewart, D. Curulla Ferré, J. Pérez-Ramírez, *ACS Catalysis* **2019**, *10*, 1133-1145.
